# Magnetic resonance imaging for the diagnosis of Parkinson’s disease

**DOI:** 10.1007/s00702-017-1717-8

**Published:** 2017-04-04

**Authors:** Beatrice Heim, Florian Krismer, Roberto De Marzi, Klaus Seppi

**Affiliations:** 1 Department of Neurology, Medical University of Innsbruck, Anichstraße 35, 6020 Innsbruck, Austria; 2 Neuroimaging Research Core Facility, Medical University Innsbruck, Innsbruck, Austria

**Keywords:** Parkinson's disease, MRT, Atypical parkinsonism, Multiple system atrophy, Progressive supranuclear palsy

## Abstract

The differential diagnosis of parkinsonian syndromes is considered one of the most challenging in neurology and error rates in the clinical diagnosis can be high even at specialized centres. Despite several limitations, magnetic resonance imaging (MRI) has undoubtedly enhanced the diagnostic accuracy in the differential diagnosis of neurodegenerative parkinsonism over the last three decades. This review aims to summarize research findings regarding the value of the different MRI techniques, including advanced sequences at high- and ultra-high-field MRI and modern image analysis algorithms, in the diagnostic work-up of Parkinson’s disease. This includes not only the exclusion of alternative diagnoses for Parkinson’s disease such as symptomatic parkinsonism and atypical parkinsonism, but also the diagnosis of early, new onset, and even prodromal Parkinson’s disease.

## Introduction

Parkinson’s disease (PD) is a slowly progressive neurodegenerative movement disorder characterized clinically by bradykinesia and additional cardinal motor features including rigidity, rest tremor and—later in the disease course—postural instability (Kalia and Lang 2015). The early differential diagnosis of degenerative parkinsonian disorders on clinical grounds can be challenging. The correct diagnosis of PD, however, is important for patient counselling and clinical research purposes. Clinico-pathological series suggest that error rates for a clinical diagnosis of PD can be as high as 24%, even at specialized centres (Hughes et al. 2002). While these studies identified atypical parkinsonian disorders (APDs) such as multiple system atrophy (MSA), progressive supranuclear palsy (PSP) and less frequently corticobasal degeneration (CBD) as the most common misdiagnoses for a clinical diagnosis of PD and vice versa, in clinically based studies other common errors relate to essential tremor (ET), drug-induced parkinsonism (DIP), and vascular parkinsonism (Rajput et al. 1991; Meara et al. 1999; Jankovic et al. 2000; Hughes et al. 2002; Schrag et al. 2002; Tolosa et al. 2006).

Because structural brain imaging using conventional MRI (cMRI) with visual assessment of T2- and T1-weighted sequences is usually normal in patients with early PD, its main role is detecting or ruling out other underlying pathologies causing parkinsonism (Mahlknecht et al. 2010). Over the past three decades, MRI has been explored as a tool to enhance diagnostic accuracy in differentiating PD from other types of parkinsonism.

This review focuses on MRI as a diagnostic tool for PD. Some parts of the text or phrases are repurposed from previous publications of the authors (Hotter et al. 2009; Mahlknecht et al. 2010).

## Techniques

Regional changes in tissue volume, signal changes on cMRI and increased deposition of iron are surrogate markers of underlying neurodegeneration and may reflect cell loss, microglial proliferation and astroglial activation. These changes can be detected by structural MRI in a qualitative way. Moreover, MRI also allows quantitative evaluation of these brain abnormalities (Rizzo et. al 2016b; Mahlknecht et al. 2010). Table 1 summarizes MR markers used to indicate different features of neurodegeneration.

**Table 1:** MR markers to detect different features of neurodegeneration

	Neurodegenerative feature				
	Neuronal/axonal loss	Myelin disruption	Gliosis	Iron content	Connectivity
T1 sequences	***Atrophy***; diameter, area, volumes, automated volume analysis				
T2 sequences	***Atrophy***		***Signal increase***	***Signal decrease***; R2 increase	
NM-MRI^a^	***Signal decrease***; volume, signal intensity				
MTI	MTR	MTR			
Diffusion imaging	MD, FA, AD, FW	FA, RD	MD, FW		Structural connectivity^b^
Iron-sensitive sequences				***Signal decrease, anatomical structures*** (DNH); different metrics^c^	
1H-MRS	NAA, NAA/Cho, NAA/Cr	Cho			
rs-fMRI					Functional connectivity^d^
ASL					Functional connectivity^e^

Qualitative markers in italic and bold; quantitative markers in recte

*AD* axial diffusivity, *ASL* arterial spin labelling, *Cho* choline, *rCBF* regional cerebral blood flow, *CMRO2* cerebral metabolic rate of oxygen consumption, *Cr* creatine, *DNH* dorsolateral nigral hyperintensity, *1H-MRS* proton magnetic resonance spectroscopy, *FA* fractional anisotropy, *FW* free water, *GRE* gradient echo sequences, *MD* mean diffusivity, *MTI* magnetization transfer imaging, *MTR* magnetization transfer ratio, *NAA N*-acetylaspartate, *NM-MRI* neuromelanin-sensitive MRI, *R2* T2 relaxation rate, R2* T2*  relaxation rate, *RD* radial diffusivity, *rs-fMRI* resting-state functional MRI, *SWI* susceptibility-weighted imaging

^a^Refer to neuromelanin-containing structures (substantia nigra and locus coeruleus)

^b^Different metrics: e.g. diffusion metrics within the tracts, number of tracks, connection probability between regions

^c^Depending on the sequence (e.g. R2* with GRE sequences; phase shift values with SWI, iron percentage with SWI, SWI signal intensities)

^d^Different metrics: e.g. correlation coefficient, integration (quantifies how signals covary between regions belonging to a particular network), small-world network indices

^e^Different metrics: e.g. rCBF or CMRO2

The introduction of high-field MRI technology with 3.0 Tesla (T) or higher field strengths has brought many advantages. The most straightforward advantage of high-field MRI is the increased signal-to-noise ratio (SNR) that scales linearly with the field strength. Increased SNR can be investigated into decreased acquisition time, increased spatial resolution or a combination of both. Spectacular anatomic delineation that is provided by high-definition scanning may improve sensitivity to smaller lesions (Lehericy et al. 2017). Furthermore, high-field MRI leads to a better grey-to-white-matter contrast, showing sharp images and smooth transitions between the different brain structures.

Recent advances in image analysis algorithms led to the development of novel approaches for automated differentiation of parkinsonian syndromes on single-patient level. These fully automated methods use support vector machine (SVM) classification and other machine-learning method-derived classification algorithms for quantitative MRI analysis including volumetric datasets (Huppertz et al. 2016; Scherfler et al. 2016), neuromelanin-sensitive MRI (NM-MRI) (Castellanos et al. 2015) and resting-state functional MRI (rs-fMRI) (Chen et al. 2015).

### Structural magnetic resonance imaging (MRI) with conventional MRI sequences

Due to its high spatial and contrast resolution, cMRI with assessment of T1-, T2-, proton density-weighted as well as T2 fluid-attenuated inversion recovery (FLAIR) sequences offers in vivo visualization of regional, disease-specific tissue alterations and certain cMRI patterns that are typical for APDs. Atrophy patterns are better demonstrated by T1-weighted images, displaying anatomical details and providing an excellent grey and white matter contrast. More recently, advanced T1 sequences were developed to improve detection of nigral changes in PD patients. These include a variety of inversion recovery images (Hutchinson and Raff 1999, 2008; Hutchinson et al. 2003; Mahlknecht et al. 2010) and a recently developed neuromelanin-sensitive T1-weighted sequence (Schwarz et al. 2011; Nakamura and Sugaya 2014; Reimao et al. 2015a). On NM-MRI, neuromelanin acts as a paramagnetic agent because of its iron-binding potential. On these images, neuromelanin-containing tissues appear as loci of high signal intensity allowing measurements of volume and concentration of neuromelanin in the substantia nigra (SN) and locus coeruleus (LC) (Nakamura and Sugaya 2014). Moreover, it seems that visual inspection of NM-MRI sequences by experienced neuroradiologists provides results comparable to quantitative analyses in the detection of SN changes in early stage PD (Reimao et al. 2015b).

T2-weighted sequences are more sensitive to changes in tissue properties; increased T2-signal reflects either degeneration, demyelination, or gliosis of the affected white matter, while a decreased T2-signal is generally restricted to the subcortical grey matter nuclei and may point toward a deposit of paramagnetic substances. The sensitivity of signal changes due to iron deposition can be increased using T2*-weighted gradient echo or susceptibility-weighted sequences. The combination of increased sensitivity to magnetic susceptibility effects attributable to iron and increased spatial resolution at higher field strengths may result in more accurate quantification of iron deposition in subcortical nuclei such as SN and striatum, which may be helpful in the discrimination of neurodegenerative parkinsonian disorders (Mahlknecht et al. 2010). Indeed, increased T2-signal seems to be better detected at higher field strengths as shown in a study using brain MRI at 0.35, 1.5, and 3.0 T in patients with MSA and PD (Watanabe et al. 2010). With increasing field strength, the occurrence of hypointensity at the dorsolateral putaminal margin increased in patients with MSA (Watanabe et al. 2010). Thus, signal abnormalities seem to be influenced by the applied magnetic field strength (Mahlknecht et al. 2010). However, field strength-related changes might result in false-positive findings. Intriguingly, in PD or healthy controls a hyperintense putaminal rim at T2-weighted images at 1.5 T has rarely been reported, whereas a hyperintense putaminal rim on T2-weighted images at 3.0 T seems to be a non-specific, common finding (Lee et al. 2005).

### Quantitative MRI

While conventional MRI sequences are generally qualitatively evaluated, quantitative evaluation of macro- and microstructural alterations as well as biochemical changes can be performed with advanced MR methodology. Advanced MR techniques include quantitative assessment of regional cerebral atrophy including MR-planimetry and -volumetry, quantitative structural MR-based techniques including diffusion imaging, magnetization transfer imaging (MTI), iron-sensitive sequences and sequences based on T1, as well as functional imaging techniques including proton magnetic resonance spectroscopy (1H-MRS), arterial spin labelling (ASL) and rs-fMRI. Moreover, new analytic methods including voxel-based analyses, machine-learning techniques and other post-processing algorithms have gained growing popularity in medical image analysis to allow quantitative evaluation of brain abnormalities.

#### Quantitative assessment of regional cerebral atrophy

Quantitative measurements of diameters, areas and volumes with region of interest (ROI) approach can be performed (Mahlknecht et al. 2010). Using an inversion pulse, the contrast of T1-weighted images can be improved as performed in a magnetization-prepared rapid acquisition with gradient echo (MPRAGE) sequence which results in high-resolution 3-D datasets, allowing more accurate quantification of volume loss (Brant-Zawadzki et al. 1992; Hotter et al. 2009). In contrast to operator-dependent segmentation techniques including region of interest (ROI) selection, voxel-wise analyses of volume differences such as voxel-based morphometry (VBM) permit an operator-independent and automated detection of significant differences in different tissue types of the whole brain involving voxel-wise statistical analysis of preprocessed structural MR images with the aid of statistical parametric mapping (Josephs et al. 2004). VBM is based on co-registration of high-resolution 3-D datasets as obtained by MPRAGE sequences, which are normalized to a study-specific template for detection of volume differences between two or more groups (Ashburner and Friston 2000). While voxel-based analyses provide group-wise comparisons of brain volume differences, fully automated segmentation software based on structural MRI such as FreeSurfer is able to measure brain volumes on an individual basis (Fischl and Dale 2000; Messina et al. 2011). This software enables automatic segmentation of the brain into multiple neuroanatomically defined regions and quantifies brain tissue volume.

There are also several attempts to assess volumes of the SN on high-field MRI in PD including multispectral structural MR imaging at 3.0 T creating a weighted mean of multiple echoes (from multiecho T1-weighted, multiecho proton density, T2-weighted, and T2-weighted FLAIR sequences) (Ziegler et al. 2013), high-resolution volumetric method based on a single pulse observation of T1 (Menke et al. 2009) and NM-MRI (Castellanos et al. 2015). More recently, approaches to investigate shapes of subcortical nuclei using T2* (Cho et al. 2011; Kwon et al. 2012) or shape analysis based on T1 imaging (Sterling et al. 2013; Menke et al. 2014; Nemmi et al. 2015) have been introduced at higher field MRI.

#### Quantitative structural MR-based techniques

*Diffusion imaging* is sensitive to the random Brownian motion of water molecules, quantified by the calculation of the apparent diffusion coefficient (ADC). Although diffusion is generally restricted alongside fibre tracts, microstructural damage might widen the space between intact fibres, increasing the mobility of water molecules and resulting in higher ADC values, respectively. Diffusion imaging measured in only one direction can lead to an underestimation of diffusion-related pathological changes because the fibre tracts are not orientated in the same direction. The trace of diffusion tensor Trace (D) is given by the average of ADCs (ADCave) measured in three orthogonal directions and is by definition independent of anisotropy (Schocke et al. 2004; Mahlknecht et al. 2010). The term diffusivity used in this review includes Trace (D), ADCave and mean diffusivity (MD). The complex neuronal architecture is organized in fibre bundles surrounded by dense myelin sheaths. This leads to a distinct anisotropy of water diffusion, which is facilitated along the direction of fibre tracts and restricted perpendicular to the fibres. While diffusion-weighted imaging (DWI) estimates water diffusion through the application of magnetic field gradient pulses, diffusion tensor imaging (DTI) requires the application of strong diffusion gradients in at least six directions (Stoessl et al. 2014), and the degree of anisotropy can be quantified with permitting calculation of fractional anisotropy (FA) (Le Bihan 2003; Hagmann et al. 2006). Diminished FA values represent tissue degeneration, either due to normal ageing or due to pathological processes as neurodegeneration. Both diffusivity and FA can be combined to form the so-called diffusion tensor, which indicates direction and extent of diffusivity with the help of a vector (Le Bihan 2003; Hagmann et al. 2006; Hotter et al. 2009). This indicates the direction and dimension of diffusivity via a vector (Le Bihan 2003; Schocke et al. 2004; Hagmann et al. 2006). Other measures of DTI include axial (or longitudinal) diffusivity (AD), which is the diffusion along the main direction of diffusion attributed to axonal damage and radial (or transverse) diffusivity (RD), which is the diffusion perpendicular to the main direction of diffusion thought to indicate myelin damage (Stoessl et al. 2014).

More recently, advanced post-processing methods to analyse diffusion imaging have been introduced. Tractography is a technique based on visually representation of neuronal fibre tracts in the brain using data collected by diffusion imaging (Stoessl et al. 2014). Tracts are reconstructed by anticipating that bundles of neuronal fibre tracts cause asymmetrical water diffusion (anisotropy) and that the main direction of the diffusion indicates the local orientation of the fibres (Mori et al. 1999; Dell’Acqua and Catani 2012; Tessitore et al. 2016).

There is a direct, but non-linear correlation between the degree of anisotropy and the number of fibres. The presence of free water (i.e. water molecules that are not restricted by the cellular environment and, therefore, do not display a directional dependence) can significantly bias diffusion indices and lead to reduced fractional anisotropy and increased mean diffusivity values. To address this issue, a bi-tensor model was introduced that separates the diffusion properties of water in brain tissue from those of water in extracellular space (Pasternak et al. 2009). Free water (FW) is water molecules that do not experience a directional dependence or other restrictions by the cellular environment (Ofori et al. 2015a, b). Although the fractional volume of FW was increased in the posterior region of the substantia nigra, the FW-corrected FA maps can be unchanged in the posterior substantia nigra of patients with PD as compared with controls (Ofori et al. 2015a, b). Neurite orientation dispersion and density imaging (NODDI) presumes an intracellular, extracellular, and cerebrospinal fluid (CSF) tissue model for each voxel. Therefore, it is able to detect the microstructure of dendrites and axons and provide data on neuronal changes suggested to be even more specific than via DTI (Zhang et al. 2012).

*MTI* is a technique which refers to interactions between protons within patterns such as myelin or cell membranes and the mobile protons of free water (Wolff and Balaban 1989; Hotter et al. 2009). Depending on the exchange rate between bound and free protons, the free water pool becomes partially saturated and a new contrast is established through radiofrequent pulses which selectively reduce the magnetization of bound water, whereas free water is unaffected. Therefore, this turns into a decrease in the free water signal as the exchange rate between free and bound water protons increases (Hotter et al. 2009). Depending on the concentration of macromolecules, which is markedly reduced in demyelinated lesions, the distinction between signal intensities with and without magnetization transfer (MT) varies (van Buchem et al. 1999). The amount of MT correlates with the myelinization degree (Rademacher et al. 1999) and axonal density (van Waesberghe et al. 1999), which can be quantified by the calculation of the magnetization transfer ratio (MTR).

*Iron-sensitive techniques* comprising T2*, susceptibility-weighted imaging (SWI), SWI phase images and quantitative susceptibility mapping (QSM) are sensitive to the presence of paramagnetic iron, which is found in the substantia nigra. Beside visual inspection of iron-sensitive images, there are also quantitative approaches to analyse sequences for iron-content detection. Relaxometry techniques use relaxation rates R2 and R2* (i.e. the reciprocal values of T2 and T2* relaxation times, whereas R2* offers a higher sensitivity compared to R2) that are reflective of the variance of the magnetic field that is generated not only by local tissue magnetic susceptibility but also by surrounding tissue susceptibility, which can be confounded by other factors such as calcium, lipid, or myelin content (Mahlknecht et al. 2010; Wang et al. 2012b; Weingarten et al. 2015; Tuite 2016). Meanwhile, SWI and QSM generated through magnitude and phase images from gradient echo MRI sequences are another means of iron quantification (Tuite 2016), reflecting susceptibility of local tissues by being less influenced by changes in water content, local water diffusion rates in inhomogeneous field and macroscopic magnetic field inhomogeneities (Du et al. 2016). Therefore, these techniques are potentially superior methods of measuring iron in vivo reflecting quantitative susceptibility of local tissues instead of the combined transverse relaxation and local field inhomogeneity indicated by R2* (Wang et al. 2012b). Other promising quantitative markers of iron imaging such as T1rho are in development (Tuite 2016).

Moreover, T1 sequences can be used to quantify SN and LC signal changes in PD patients, which include a variety of inversion recovery images (Mahlknecht et al. 2010) and NM-MRI (Nakamura and Sugaya 2014).

#### Functional imaging techniques

*rs-fMRI* is a method to assess functional brain imaging to evaluate regional interactions when a subject is not performing an explicit task and visualizes functional brain connectivity changes (Biswal 2012; Buckner et al. 2013).

*ASL* is a magnetic resonance imaging technique for measuring tissue perfusion using magnetically labelled protons in arterial blood water as an endogenous tracer (Wolf and Detre 2007). ASL is non-invasive and able to quantitatively measure tissue perfusion. Recent technical advances have increased its sensitivity and also extended its potential applications (Petcharunpaisan et al. 2010).

*Magnetic resonance spectroscopy* (*MRS*) is a non-invasive technique to measure and quantify spectra of many biologically important metabolites. 1H-MRS, the most used in clinical practice, can measure levels of specific hydrogen-containing compounds in vivo. In vivo proton 1H-MRS visualizes signals from carbon-bound, non-exchangeable protons, showing the highest information density in the spectral region from 1 to 5 ppm (Seppi and Schocke 2005). Principal metabolites detected by 1H-MRS include *N*-acetylaspartate (NAA) as an indirect expression of the integrity of neurons, choline-containing compounds (Cho; such as metabolites involved in phospholipid membrane synthesis) as markers for glial activity, creatine [including phosphocreatine (Cr), whose peak is relatively stable and commonly used as a concentration internal reference] as a marker for energy metabolism, lactate as an indicator for anaerobic glycolysis detected under pathologic conditions as well as different other metabolites. The NAA/Cr ratio is a metabolic marker that reflects function and integrity of neurons and axons in the brain. A decrease of this ratio indicates neuronal or axonal dysfunction (Firbank et al. 2002; Schocke et al. 2003; Seppi and Schocke 2005; Hotter et al. 2009; Rizzo et al. 2016b). MRSI is an advanced 1H-MRS technique, which acquires spectra simultaneously over a large brain region from multiple voxels during the same sequence allowing not only the spatial location of the voxels to be changed (without loss of quality) by sub-voxel shifts during post-processing, but allowing also individual voxels to be aligned with anatomical features and allowing an absolute quantification of metabolites such as NAA to be performed (Guevara et al. 2010). As the most commonly used standards (Cr and Cho) seem to vary in concentration, quantitative analysis techniques show advantages compared with alternative ratio-based methods (Esterhammer et al. 2010).

#### Multimodal imaging

Multimodal imaging is an approach to fuse information from different modalities. Multimodal imaging studies in PD showed that combinations of different methods sensitive to complementary tissue characteristics may provide better distinction than single techniques (Menke et al. 2009; Peran et al. 2010; Du et al. 2011; Kassubek and Muller 2016 ; Esterhammer et al. 2015). Combined R2* and diffusion tensor imaging changes in the substantia nigra in Parkinson’s disease as well as (Du et al. 2011) variable combinations of volumetry, R2*, MD, or FA, (Menke et al. 2009; Peran et al. 2010; Du et al. 2011) have been used in PD and, more recently, a multi-contrast study assessed iron deposition using SWI in regions of the SN pars compacta (SNc) defined by NM-MRI (Langley et al. 2016).

## Exclusion of alternative diagnoses

Structural MRI with conventional MR sequences is usually normal in early PD patients limiting its application in clinical routine for the detection of early PD. Recent studies, however, identified imaging correlates of underlying neuropathology in PD patients through advanced MRI techniques. These imaging abnormalities will be discussed in detail later in this review. Nevertheless, cMRI was repetitively shown to be useful in discriminating PD from APDs such as MSA and PSP. Latter are characterized by disease-specific atrophy patterns and signal intensity changes. In addition, current operational diagnostic criteria require the exclusion of symptomatic causes of parkinsonism in the work-up of patients with PD (Gibb and Lees 1988).

### Exclusion of symptomatic parkinsonism

**Fig. 1:**
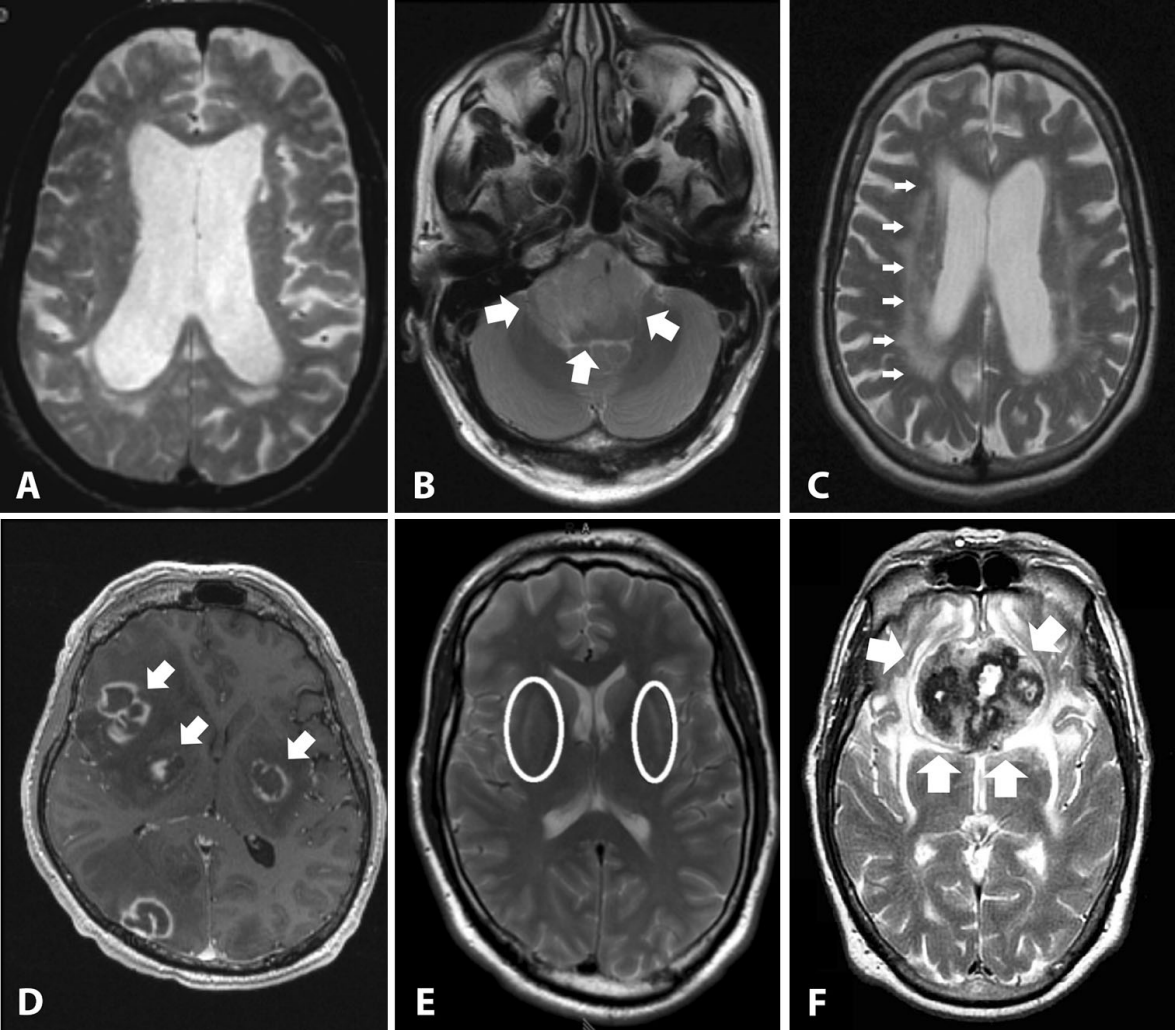
Secondary causes of parkinsonism. This figure shows examples of secondary causes of parkinsonism. **a** Normal pressure hydrocephalus with disproportionally dilated lateral ventricles and periventricular hyperintensities suggesting transependymal flow on an axial T2 image. **b** Brainstem tumor mass (glioma) on an axial T2 image. **c** Severe leucencephalopathy with multiple  white matter lesions on an axial T2 image in a patient with vascular parkinsonism. **d** Central nervous system toxoplasmosis with contrast enhanced lesions (also in the basal ganglia) on a contrast-enhanced axial T1 image in a HIV-positive patient. **e** Hypoxic basal ganglia lesions (putaminal signal increases on an axial T2 image) after carbon monoxide poisoning. **f** Olfactory meningioma as an example for a frontal space-occupying lesion on an axial T2 image. Modified from Neuroimaging of Movement Disorders, Structural MRI in Idiopathic Parkinson Disease and Parkinsonism, Volume 44 of the series Current Clinical Neurology, 2013, pp 105-128, Mueller C et al., with permission of Springer

Structural brain imaging using cMRI with visual assessment of T2- and T1-weighted sequences including contrast-enhanced T1 imaging is usually normal in patients with early PD; thus, its traditional role is the detection/exclusion of other underlying basal ganglia or brainstem pathologies (Hotter et al. 2009; Mahlknecht et al. 2010). These include vascular, space-occupying or demyelinating lesions within the basal ganglia or brainstem, drug- or toxic-induced parkinsonism, e.g. due to manganism, or neurodegeneration with brain iron accumulation (NBIA), normal pressure hydrocephalus, or infectious causes (see Table 2; Fig. 1) (Hotter et al. 2009; Mahlknecht et al. 2010). Typical MR findings in patients with symptomatic parkinsonism are summarized in Table 2.

**Table 2:** MRI findings for differential diagnosis in symptomatic parkinsonism

Entity	Typical MRI findings (may vary)
Vascular parkinsonism	Lacunar infarctions in the basal ganglia, frontal lobe infarctions, subcortical microangiopathic lesions with diffuse periventicular signal alterations
Normal pressure hydrocephalus	Enlargement of lateral cerebral ventricles, ballooning of anterior horn of lateral ventricle, periventricular T2 signal alterations
Toxic-induced parkinsonism	
Manganese	Hyperintensities in the globus pallidus, increasing signals in T1-weighted sequences in the striatum and SN
Ephedron (methcathinone)	Increased bilateral and symmetric T1-signal intensity in the globus pallidus and hyperintensities in the SN, no signal abnormalities on T2-weighted images
Carbon monoxide	Transient bilateral symmetric lesions in the globus pallidus with hyperintensities in T2-weighted images
Cyanide	Symmetric hyperintense signal changes in the globus pallidus, putamen, caudate nucleus, and white matter areas in T2-weighted images and FLAIR sequences, lesions in the basal ganglia displaying T1 signal increase with contrast enhancement
Methanol	T2 signal increase and T1 signal decrease in the area of the putamen
Huntington’s disease (Westphal variant)	Progressive bilateral atrophy of the striatum and caudate nucleus with enlarged anterior horn of lateral ventricle: in the later course widespread atrophy throughout the cortex;
Wilson’s disease	Atrophy of the midbrain, brain stem, and cerebellum; marked T2 hypointensity in the globus pallidus, symmetric T2 hyperintensity in the striatum, lateral thalamus, white matter, and dorsal brain stem; “face of the giant panda”: T2-weighted axial MRI with normal signal at the red nuclei (eyes) and lateral aspects of the SN (ears) with signal increase at the tegmentum and hypointense superior colliculi
Neurodegeneration with iron accumulation (NBIA)	
Panthothenate kinase-associated neurodegeneration (PKAN)	Decreased signal in T2-weighted sequences in the globus pallidus, putamen, caudate nucleus, and thalamus; „eye of the tiger“ sign: high signal in the center of the globus pallidus and T2 hypointensity of the surrounding area
Aceruloplasminaemia and neuroferritinopathy	T2 hypointensity in the globus pallidus, SN, striatum, thalamus, and dentate nucleus
Cerebral masses	Characteristic structural imaging according to the CNS tumours’ entity
Multiple sclerosis	T1‐weighted hypointense lesions (“black holes”) and hyperintensities in T2-weighted sequences in the SN and basal ganglia

### Exclusion of atypical parkinsonism

The umbrella term *atypical parkinsonism* covers neurodegenerative disorders that feature rapidly progressive parkinsonism together with additional, often debilitating symptoms that are uncharacteristic for idiopathic PD. MSA, PSP and CBD fall under this category. Neuropathologically, PD and MSA share prominent alpha-synuclein inclusion pathology. Intriguingly, inclusion bodies in MSA patients are predominantly seen in oligodendrocytes, whereas Lewy bodies are mostly seen in the cytoplasm of neurons. In contrast to these disorders, PSP and CBD are considered to be tauopathies with 4-repeat tau protein accumulation. For adequate patient counselling, it is important to recognize these atypical disorders since the natural course of these disorders and treatment options are different from PD. In addition, to reduce between-subject heterogeneity in interventional trials, early and accurate diagnosis is at utmost importance. However, on clinical grounds, degenerative parkinsonian disorders can be indistinguishable from one another in early disease stages and, therefore, additional investigations such as MRI may become necessary to correctly diagnose patients with atypical parkinsonism (Mahlknecht et al. 2010; Poewe et al. 2017).

#### Structural MRI with conventional MRI sequences

Using cMRI at 1.5 T with T1-, T2-, and PD-weighted sequences with its high spatial and contrast resolution, it is possible to show changes in the basal ganglia, in cortical or infratentorial structures to distinguish between atypical parkinsonism and PD. MR scanners with 1.5 T field strengths are the most commonly used technique for which most data are obtainable in patients with atypical parkinsonism. Therefore, when discussing signal changes, the authors refer to 1.5 T field strengths, unless otherwise stated.

**Fig. 2:**
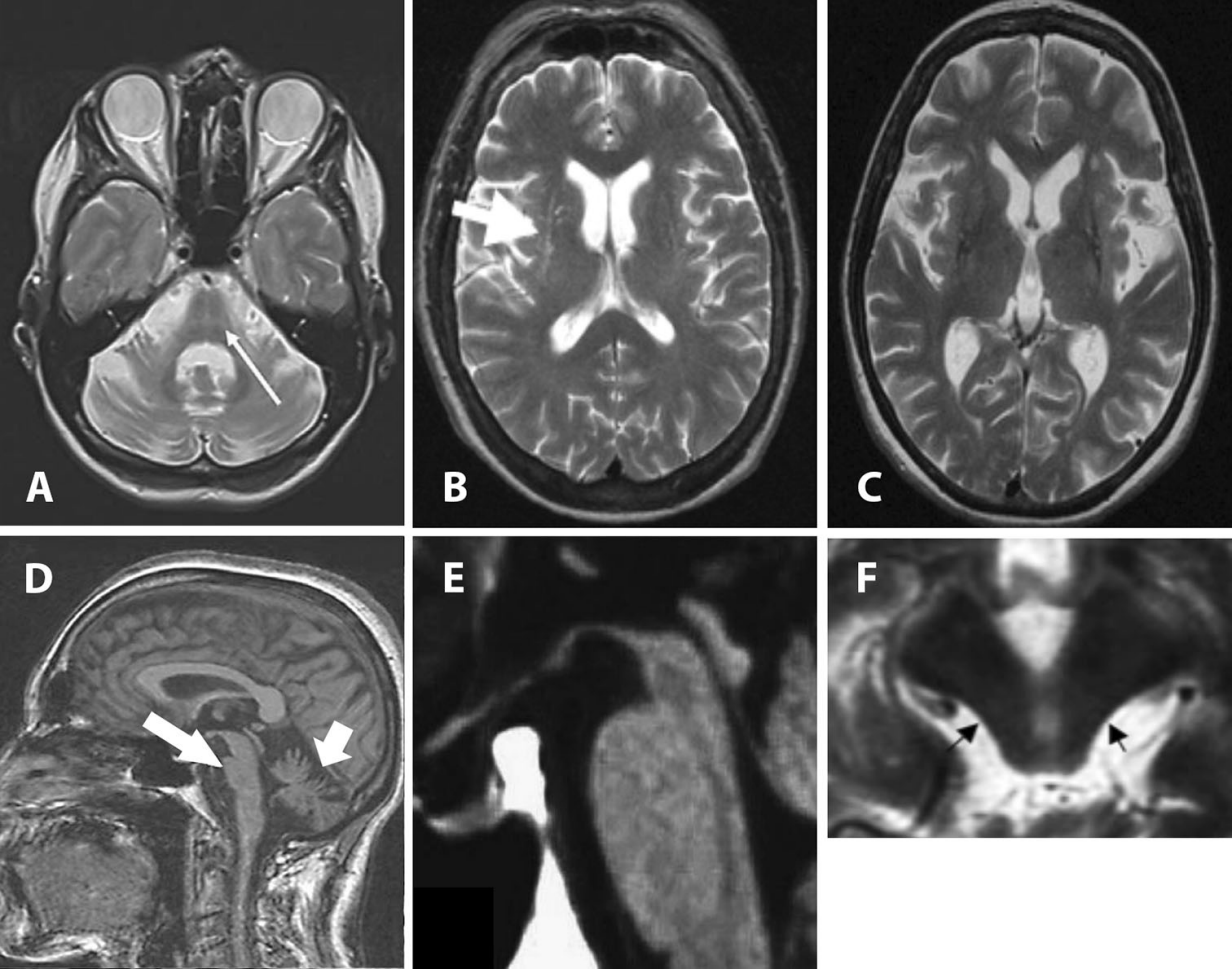
This figure illustrates a selection of MRI features that were shown to be typical findings in atypical parkinsonian disorders. **a** The hot cruss bun sign on an axial T2 image in a patient with multiple system atrophy (MSA). **b** Putaminal atrophy with the putaminal hyperintense rim (arrow) on an axial T2 image in a patient with MSA. **c** Putaminal atrophy with putaminal hypointensity on an axial T2 image in a patient with MSA. **d** Atrophy of the pons and the cerebellum on a midsagittal T1 image as a common finding in MSA reflecting olivopontocerebellar atrophy. **e** The hummingbird sign (atrophy of the rostral midbrain tegmentum) on a midsagittal T1 image and **f** the morning glory flower sign (concavity of the lateral margin of the tegmentum) on an axial T2 image reflecting midbrain atrophy in progressive supranuclear palsy patients. Modified from Neuroimaging of Movement Disorders, Structural MRI in Idiopathic Parkinson Disease and Parkinsonism, Volume 44 of the series Current Clinical Neurology, 2013, pp 105-128, Mueller C et al., with permission of Springer

The most striking imaging features in MSA are putaminal atrophy, hypointensity of the putamen, and “slit-like” marginal hyperintensity (hyperintense putaminal rim) in T2-weighted sequences as well as infratentorial abnormalities including atrophy of the lower brainstem, pons, medulla oblongata, inferior olives, middle cerebellar peduncle (MCP), and cerebellum as well as hyperintensities in the pons, MCP, and cerebellum. Some of these changes are illustrated in Fig. 2. Even though putaminal atrophy seems to be quite specific to differentiate MSA and PD, the hyperintense putaminal rim sign may also occur in PD patients (Bhattacharya et al. 2002; Schocke et al. 2002; Seppi et al. 2006a, b). Generally, sensitivity of hypointense signal alterations can be improved by modifying relaxation contrast using T2*-weighted gradient echo (GE) sequences (Kraft et al. 2002; Righini et al. 2002; von Lewinski et al. 2007; Sakurai et al. 2010; Wadia et al. 2013). Moreover, signal abnormalities seem to be influenced by the applied magnetic field strength (Mahlknecht et al. 2010). Indeed, with increasing field strength the appearance of putaminal hypointensity seems to increase in patients with MSA (Watanabe et al. 2010). Moreover, in PD or healthy controls a hyperintense putaminal rim at T2-weighted images at 1.5 T has rarely been reported, whereas a hyperintense putaminal rim on T2-weighted images at 3.0 T seems to be a non-specific, common finding (Lee et al. 2005). It has to be considered that increased sensitivity to signal abnormalities with higher field strengths might result in false-positive findings for the differentiation of MSA from PD (Mahlknecht et al. 2010). Furthermore, pontine signal alterations resembling a pattern designated as “hot cross bun sign” are highly suspicious for MSA, but are also found in non-degenerative parkinsonism and in spinocerebellar ataxia (SCA) (Muqit et al. 2001; Hotter et al. 2009; Lee et al. 2009).

Specific brain MRI findings associated with PSP include atrophy of the midbrain with enlargement of the third ventricle, tegmental atrophy and an abnormal superior profile of the midbrain, signal increase in the midbrain and in the inferior olives, as well as frontal and temporal lobe atrophy (Savoiardo et al. 1994; Soliveri et al. 1999; Schrag et al. 2000; Warmuth-Metz et al. 2001; Savoiardo 2003; Righini et al. 2004; Oba et al. 2005; Paviour et al. 2005, 2006a, b; Seppi and Schocke 2005; Barsottini et al. 2007; Slowinski et al. 2008; Hotter et al. 2009; Agosta et al. 2010; Mahlknecht et al. 2010). Moreover, visual assessment of atrophy of the superior cerebellar peduncle (SCP) has been shown to distinguish PSP patients from healthy controls and patients with other parkinsonian syndromes including MSA and PD with a sensitivity of 74% and a specificity of 94% (Paviour et al. 2005).

Overall, the above-mentioned MRI abnormalities demonstrate high specificity for distinguishing MSA or PSP from PD and healthy controls. However, specificity of the putaminal changes is insufficient to differentiate MSA from other forms of atypical parkinsonism. Sensitivity of the characteristic findings is suboptimal, particularly in early stages of the disease, and literature reports are inconsistent with a broad range of sensitivity values being reported. Indeed, about 60% of patients with the parkinsonian variant of MSA (MSA-P) had neither putaminal nor infratentorial changes within 2 years from disease onset as reported by a study, in which MRI findings at the first hospital visit were analysed for 139 patients with MSA including 54 patients with MSA-P (Watanabe et al. 2002). Interestingly, a recent study on 48 neuropathologically confirmed cases with neurodegenerative parkinsonism found that radiological assessment of MRI was correct in 16 of 22 (73%) PSP cases and 10 of 13 (77%) MSA cases with no PSP case misclassified as MSA or vice versa suggesting that the above-mentioned MR abnormalities are specific for MSA and PSP if these MR abnormalities are inspected and evaluated together (Massey et al. 2012). Moreover, this study showed that characteristic findings may not be present even at autopsy.

Investigating the role of cMRI in the diagnostic work-up of CBD, there are only few studies available, showing cortical—especially frontoparietal—atrophy, which tends to be asymmetric, putaminal hypointensity as well as hyperintense signal changes in the motor cortex or subcortical white matter on T2-weighted images (Hauser et al. 1996; Soliveri et al. 1999; Schrag et al. 2000; Savoiardo 2003; Josephs et al. 2004). Yet, these abnormalities seem to be of barely diagnostic relevance for CBD (Schrag et al. 2000; Josephs et al. 2004; Hotter et al. 2009). Intriguingly, a review of 40 autopsy cases with life-time diagnosis of a CBD showed that neither cortical nor corpus callosum atrophy nor subcortical and periventricular white matter signal changes on MRI were specific to CBD but showed similar patterns in the patients with other neurodegenerative diseases (Josephs et al. 2004).

#### Quantitative MRI

Tables 3 and 4 summarize the most relevant studies on quantitative MRI to determine atypical parkinsonism.

**Table 3:** Diagnostic accuracy of the quantitative assessment of regional cerebral atrophy including MR-planimetry, -volumetry, and automated methods for quantitative MRI analysis for the diagnosis of APD

References	Cohort size	Magnetic field	Main results	Discriminator	Accuracy, %
Schulz et al. (1999)	MSA-P 12/MSA-C 17/PD 11/PSP 6/HC 16	1.5 T	↓ Mean striatal and brainstem volumes in MSA-P, MSA-C, and PSP ↓ Cerebellar volume in MSA-C and MSA-P Volumes were TIV-corrected Patients with PD could not be separated from HC and patients with MSA-P could not be separated from patients with PSP	Discriminant function including volumes of brainstem, caudate nucleus, putamen, and cerebellum (stepwise linear discrimination)^a^	Overall correct classification 65% 91% of APD and 89% of non-APD classified correctly 75% of PD, 36% of HC, 67% of MSA-P, 76% of MSA-C and 50% of PSP classified correctly
					Se 76 (MSA-C) Sp 100 (vs. PD), 82 (vs. HC), 100 (vs. PSP)
Cordato et al. (2002)	PSP 21/PD 17/HC 23	1.5 T	↑ Ventricular volume, ↓ whole brain and frontal GM volumes in PSP vs. PD and HC	Frontal GM volume	Se 95 (PSP) Sp 91 (vs. other groups)
Groschel et al. (2004)	PSP 33/CBS 18/HC 22 (including 8 PSP and 7 CBD with a post-mortem confirmed diagnosis)		↓ Brainstem volume (>midbrain) in PSP vs. CBS and HC ↓ Parietal and occipital lobes volumes (>white matter) in CBS vs. PSP and HC ↓ Area of CC in CBS vs. PSP and HC Volumes were TIV-corrected	Discriminant function including midbrain, parietal WM, temporal GM, brainstem, frontal WM, and pons volumes	Overall correct classification 84% 95% of HC, 76% of CBS, 83% of PSP classified correctly
Paviour et al. (2005)	PSP 19/MSA 10/PD 12/HC 12	1.5 T	↓ SCP volume in PSP vs. MSA, PD, and HC Volumes were TIV-corrected	SCP volume	Se 74 (PSP) Sp 77 (vs. other groups)
Paviour et al. (2006b)	MSA-P 9/PSP 18/PD 9/HC 18	1.5 T	↓ Midbrain and SCP volumes in PSP compared to MSA-P, PD and HC ↓ Frontal volume in PSP compared to PD and HC, but not to MSA-P Significant greater third ventricle in PSP compared to HC, but not to MSA-P and PD ↓ Cerebellar, pontine, and posterior inferior volumes in MSA-P compared to PD and HC ↓ Midbrain volume in MSA-P compared to HC No significant differences between PD and HC	Midbrain, SCP, frontal, third ventricle, and whole brain volumes	Se 89 (PSP)^b^ Sp 97 (vs. MSA-P, PD, and HC)
				SCP, midbrain, pons, and cerebellar volumes	Se 94 (PSP)^b^ Sp 89 (vs. MSA-P)
Cosottini et al. (2007)	PSP 15/MSA-P 7/HC 14	1.5 T	↓ m_d_ in PSP vs. HC, but is not significantly different between PSP and MSA-p patients ↓ m_a_ in PSP vs. MSA-P and HC ↓ Midbrain volume in PSP patients vs. HC, but does not differ with respect to MSA-P ↓ p_a_ in MSA-P vs. PSP and HC ↓ m_a_/p_a_ ratio is in PSP vs. MSA-P and HC ↑ m_a_/p_a_ ratio in MSA-P vs. HC	m_d_	Se 60 (PSP vs. MSA-P and HC) Sp 95 (vs. MSA-P and HC) AUC = 0.81 Se 60 (PSP vs. MSA-P) Sp 86 (PSP vs. MSA-P) AUC = 0.62 (PSP vs. MSA-P)
				m_a_	Se 100 (PSP vs. MSA-P and HC) Sp 90 (PSP vs. MSA-P and HC) AUC = 0.99 Se 87 (PSP vs. MSA-P) Sp 100 (PSP vs. MSA-P) AUC = 0.97
				p_a_	Se 73 (PSP vs. MSA-P and HC) Sp 62 (PSP vs. MSA-P and HC) AUC = 0.54 Se 100 (PSP vs. MSA-P) Sp 86 (PSP vs. MSA-P) AUC = 0.96
				m_a_/p_a_ ratio	Se 87 (PSP vs. MSA-P and HC) Sp 100 (PSP vs. MSA-P and HC) AUC = 0.96 Se 100 (PSP vs. MSA-P) Sp 100 (PSP vs. MSA-P) AUC = 1.00
				Midbrain volume	Se 87 (PSP vs. MSA-P and HC) Sp 76 (PSP vs. MSA-P and HC) AUC = 0.83 Se 87 (PSP vs. MSA-P) Sp 71 (PSP vs. MSA-P) AUC = 0.77
Lee et al. (2013b)	PD 29/PSP 13/MSA-P 15/HC 21	3.0 T	↓ Volume of caudate, putamen, globus pallidus, and thalamus in PSP and MSA-P vs. PD and HC ↓ Volume of globus pallidus in PSP vs. MSA-P ↓ Volume of putamen in MSA-P vs. PSP ↑ R2* in the putamen in MSA-P vs. PD and HC ↑ R2* in globus pallidus and caudate in PSP vs. PD and HC ↑ R2* in caudate nucleus in PSP vs. MSA-P	Putaminal volume	AUC = 0.83 (MSA-P vs. PD and PSP)
				Volume of globus pallidus	AUC = 0.86 (PSP vs. PD and MSA-P)
Baudrexel et al. (2014)	PD 13/PSP 8/MSA-P 11/HC 6	3.0 T	↓ Putaminal volume in MSA-P vs. PD, PSP, and HC	Putaminal volume	Se 54.5 (MSA-P) Sp 100 (vs. other groups) AUC = 0.84
Zanigni et al. (2016)	PSP-RS 23/PD 42	1.5 T	↑ Sagittal MCP_d_/coronal SCP_d_ ratio, p_a_/m_a_ratio, and MRPI in PSP-RS vs. PD ↓ Sagittal MCP_d_, coronal SCP_d_, p_a_, and m_a_ in PSP-RS vs. PD ↑ DTI MD in SCP, thalamus, putamen, globus pallidus, parieto-occipital WM, pre-frontal WM, right brain emisphere, left brain hemisphere, posterior fossa, brainstem, and in cerebellar hemispheres in PSP-RS vs. PD ↓ DTI FA in SCP, midbrain, parieto-occipital WM, pre-frontal WM, right brain hemisphere, left brain hemisphere, posterior fossa, and in brainstem in PSP-RS vs. PD ↓ Volume of brainstem, globus pallidus, putamen and thalamus in PSP-RS vs. PD ↑ Volume of lateral ventricles in PSP-RS vs. PD	m_a_	Se 96 (PSP-RS) Sp 98 AUC = 0.99
				p_a_/m_a_-ratio	Se 96 (PSP-RS) Sp 90 AUC = 0.97
				MRPI	Se 87 (PSP-RS) Sp 93 AUC = 0.95
				Coronal SCP_d_	Se 81 (PSP-RS) Sp 74 AUC = 0.84
				Pons area	Se 87 (PSP-RS) Sp 74 AUC = 0.82
				MD SCP	Se 70 (PSP-RS) Sp 98 AUC = 0.88
				MD pre-frontal WM	Se 90 (PSP-RS) Sp 69 AUC = 0.86
				MD thalamus	Se 70 (PSP-RS) Sp 86 AUC = 0.84
				MD putamen	Se 80 (PSP-RS) Sp 71 AUC = 0.82
				MD posterior fossa	Se 80 (PSP-RS) Sp 83 AUC = 0.90
				MD cerebellar hemispheres	Se 80 (PSP-RS) Sp 74 AUC = 0.86
				MD right brain hemisphere	Se 75 (PSP-RS) Sp 90 AUC = 0.88
				MD left brain hemisphere	Se 65 (PSP-RS) Sp 95 AUC = 0.87
				MD brainstem	Se 85 (PSP-RS) Sp 67 AUC = 0.80
				FA SCP	Se 75 (PSP-RS) Sp 80 AUC = 0.82
				FA parieto-occipital WM	Se 75 (PSP-RS) Sp 80 AUC = 0.82
				FA right brain hemisphere	Se 75 (PSP-RS) Sp 90 AUC = 0.88
				FA left brain hemisphere	Se 65 (PSP-RS) Sp 95 AUC = 0.87
				FA posterior fossa	Se 83 (PSP-RS) Sp 75 AUC = 0.80
				Volume thalamus	Se 73 (PSP-RS) Sp 90 AUC = 0.83
				Volume putamen	Se 93 (PSP-RS) Sp 70 AUC = 0.83
				Volume globus pallidus	Se 93 (PSP-RS) Sp 60 AUC = 0.81
Oba et al. (2005)	PSP 21/MSA-P 25/PD 23/HC 31	1.5 T	↓ m_a_ in PSP vs. PD and MSA-P ↓ p_a_ in MSA-p vs. PD and PSP ↓ In m_a_/p_a_-ratio in PSP vs. PD and MSA-P	m_a_	Se 100 (PSP) Sp 91 (PSP vs. non-PSP)
				m_a_/p_a_-ratio	Se 100 (PSP) Sp 100 (PSP vs. non-PSP)
Gama et al. (2010)	PSP 20/MSA-P 8/MSA-C 11/PD 21/	1.5 T	↓ m_a_ in PSP and MSA-P vs. PD, in PSP vs. MSA-C and MSA-P, and in MSA-P vs. MSA-C ↓ p_a_ in PSP, MSA-P, and MSA-C vs. PD, and in MSA-C and MSA-P vs. PSP ↓ MCP_d_ in PSP, MSA-C, and MSA-P vs. PD, and in MSA-C vs. PSP ↓ SCP_d_ in PSP and MSA-C vs. PD, and in PSP vs. MSA-C and MSA-P	m_a_	Se 95 (PSP)^b^ Sp 97 (PSP vs. other groups)
				SCP_d_	Se 80 (PSP) Sp 100 (vs. other groups)
Quattrone et al. (2008)	MSA-P 19/PD 108/PSP 33/HC 50	1.5 T	↓ m_a_ and SCP_d_ in PSP compared to PD, MSA-P, and HC with some overlap of values between groups ↑ p_a_/m_a_ and MCP_d_/SCP_d_ ratios in PSP compared to PD, MSA-P, and HC with some overlap of values between groups ↑ MRPI in PSP compared to PD, MSA-P, and HC without any overlaps values among groups	p_a_/m_a_ ratio	Se 95 (PSP)^b^ Sp 97 (vs. PD, MSA-P, and HC)
				MCP_d_/SCP_d_ratio	Se 90 (PSP)^b^ Sp 94 (vs. PD, MSA-P, and HC)
				MRPI	Se 100 (PSP)^b^ Sp 100 (vs. PD, MSA-P, and HC)
Hussl et al. (2010)	MSA-P 26/PSP 22/PD 75	1.5 T	↓ m_a_/p_a_-ratio and ↑ MRPI values in PSP compared to MSA-P, PD, and HC	m_a_/p_a_ ratio	Se 64 (PSP)^b^ Sp 92 (vs. non-PSP), 95 (vs. PD), 85 (vs. MSA-P)
				MRPI	Se 82 (PSP)^b^ Sp 80 (vs. non-PSP), 76 (vs. PD), 92 (vs. MSA-P)
Longoni et al. (2011)	PSP-RS 10/ PSP-P 10/ PD 25/HC 24	1.5 T	↑ p_a_/m_a_ ratio in PSP-RS and PSP-P vs. PD ↑ MRPI in PSP-RS and PSP-P vs. PD	p_a_/m_a_ ratio	Se 90 (PSP-RS vs. PD) Sp 96 (PSP-RS vs. PD) Se 60 (PSP-P vs. PD) Sp 96 (PSP-P vs. PD)
				MRPI	Se 100 (PSP-RS vs. PD) Sp 92 (PSP-RS vs. PD) Se 70 (PSP-P vs. PD) Sp 68 (PSP-P vs. PD)
Morelli et al. (2011a)	81 with clinically uncertain parkinsonism (of which *n* = 15 developed PSP and *n* = 11 patients developed MSA)	1.5 T	↑ MRPI in clinically uncertain parkinsonism in those patients developing PSP at follow-up compared to the other patients MRPI showed a higher accuracy in predicting PSP (92.9%) than clinical features	MRPI	Se 100 (PSP) Sp 90(PD and MSA)
Nigro et al. (2016)	PSP 88/PD 234/HC 117	1.5 T	↑ MRPI in PSP vs. PD and controls No statistical differences between automated and manual MRPI values in all groups	Automated MRPI	Se 93 (PSP) Sp 97 (vs. PD), 94 (vs. HC)
				Manual MRPI	Se 90 (PSP) Sp 100 (vs. PD), 94 (vs. HC)
		3.0 T		Automated MRPI	Se 97 (PSP) Sp 97 (vs. PD), 97 (vs. HC)
				Manual MRPI	Se 100 (PSP) Sp 100 (vs. PD) 100 (vs. HC)
Mangesius et al. (2017) (manuscript in preparation)	81 clinically uncertain parkinsonism (including *n* = 15 who developed PSP and *n* = 11 who developed MSA)	1.5 T	m_d_^c^, m_a_, m_a_/p_a_-ratio, m_d_/p_d_-ratio^c^ and MRPI showed high overall diagnostic accuracy and specificity (over 80%) in distinguishing PSP from non-PSP neurodegenerative parkinsonism	m_d_	Se 89 (PSP) Sp 91 (vs. other groups), 91 (vs. PD), 92 (vs. MSA)
				m_a_	Se 68 (PSP) Sp 87 (vs. other groups), 88 (vs. PD), 85 (vs. MSA)
				m_d_/p_d_-ratio	Se 87 (PSP) Sp 94 (vs. other groups), 94 (vs. PD), 96 (vs. MSA)
				m_a_/p_a_-ratio	Se 89 (PSP) Sp 85 (vs. other groups), 82 (vs. PD), 95 (vs. MSA)
				MRPI	Se 87 (PSP) Sp 86 (vs. other groups), 82 (vs. PD), 100 (vs. MSA)
Nicoletti et al. (2006a)	MSA 16 (MSA-P 13, MSA-C 3)/PD 26/HC 14	1.5 T	↓ Average MCP_d_ in MSA compared to PD and HC using T1-weighted sequences	MCP_d_	Se 100 (MSA) 100 (vs. PD and HC)
Massey et al. (2013)	PSP 21/PD 10/MSA 10/HC 21.	3.0 T	↓ m_d_^c^ and reduced m_d_/p_d_-ratio^c^ in PSP compared to MSA-P, PD, and HC	m_d_	Se 90 (PSP) Sp 100 (vs. PD, MSA. HC)
				m_d_/p_d_-ratio	Se 86 (PSP) Sp 100 (vs. PD, MSA. HC)
Kim et al. (2015)	PD 82/PSP 29	3.0 T	m_d_^d^ and m_a_/p_a_ ratio discriminated PSP from PD with similar discriminatory power MRPI showed lower discriminatory power	m_d_	Se 86 (PSP) Sp 54 AUC = 0.76
				m_a_/p_a_ ratio (Cosottini method) (Cosottini et al. 2007)	Se 62 (PSP) Sp 76 AUC = 0.75
				m_a_/p_a_ ratio (Oba method) (Oba et al. 2005)	Se 72 (PSP) Sp 64 AUC = 0.75
				MRPI	Se 93 (PSP-RS) Sp 43 AUC = 0.69
Moller et al. (2017)	PD 204/PSP 106/MSA-C 21/MSA-P 60/HC 73	1.5 and 3.0 T	↓ m_a_ in PSP vs. all other groups ↓ p_a_ in MSA-C, MSA-P, and PSP vs. PD and HC. ↓ m_a_/p_a_ in PSP vs. all other groups ↑ m_a_/p_a_ in MSA-C and MSA-P vs. PD and PSP	m_a_	AUC = 0.90 (PSP vs. PD) Se 75, Sp 82, AUC = 0.85 (PSP vs. MSA-P)
				m_a_/p_a_	Se 76, Sp 80, AUC = 0.84 (PSP vs. PD) Se 76, Sp 80, AUC = 0.89 (PSP vs. MSA-P)
				MRPI	Se 64, Sp 64, AUC = 0.75 (PSP vs. PD) Se 73, Sp 60, AUC = 0.80 (PSP vs. MSA-P)
Automated methods for quantitative MRI analysis					
Huppertz et al. (2016)	PD 204, PSP 106, MSA-C 21, MSA-P 60	1.5 and 3.0 T	Fully automated brain volumetry combined with SVM classification allowed for automated differentiation on single-patient level Volume changes of midbrain, basal ganglia, and cerebellar peduncles had the largest relevance for classification	Atlas-based voxel-based volumetry combined with SVM classification	Classifications between the groups resulted in balanced diagnostic accuracies ≥80%
Scherfler et al. (2016)	PSP 30/MSA 40/PD 40 of whom 40 presented with a clinically uncertain parkinsonism Data were split into a training (*n* = 72) and a test set (*n* = 38)	1.5 T	Volume segmentation of subcortical brain regions followed by a machine-learning method-derived classification algorithm (i.e. C4.5 decision tree algorithm) Most discriminative regions include the volume of the midbrain, followed by cerebellar GM and putamen Diagnostic accuracy of the fully automated method for quantitative MRI analysis was 97% for the separation of PD vs. MSA or PSP, by contrast to the clinical diagnostic accuracy of 63% based on validated clinical consensus criteria at the time of MRI	Midbrain, putaminal and cerebellar GM volume	Se 90 (PSP), 100 (MSA), 100 (PD) Sp 100

*APD* atypical parkinsonian disorders, Se sensitivity, *Sp* specificity, *AUC* area under the curve; ↑ significant higher, ↓ significant lower, *T* tesla, *MSA* multiple system atrophy, *MSA-P* parkinsonian variant of MSA, *MSA-C* cerebellar variant of MSA, *PD* Parkinson’s disease, *PSP* progressive supranuclear palsy, *HC* healthy controls, *MRI* magnetic resonance imaging, *VBM* voxel-based morphometry, *CC* corpus callosum, *WM* white matter, *GM* grey matter, *MCP* middle cerebellar peduncle, *SCP* superior cerebellar peduncle, *MRPI* magnetic resonance parkinsonism index, *m*_*d*_ midbrain diameter, *m*_*a*_ midbrain area, *p*_*d*_ pontine basis diameter, *p*_*a*_ pontine area, *SCP*_*d*_ superior cerebellar peduncle diameter, *MCP*_*d*_ middle cerebellar peduncle diameter, *m*_*d*_ */p*_*d*_ *-ratio* m_d_ to p_d_ ratio, *m*_*a*_ */p*_*a*_ *-ratio* m_a_ to p_a_-ratio, *p*_*a*_ */m*_*a*_ *ratio* p_a_ to m_a_ ratio, *MCP*_*d*_ */SCP*_*d*_ MCP_d_ to SCP_d_ ratio, *TIV* total intracranial volume, *SVM* support vector machine, R2*  T2* relaxation rate

^a^No PD patient was classified as having MSA-P or vice versa; however, three of the PD patients were classified as having MSA-C or PSP; discrimination of patients with MSA from PSP was poor

^b^Diagnostic accuracy values for planimetric measurements of regional brain structures are highest in PSP; therefore, sensitivity values were primarily given for PSP

^c^These diameters were obtained by placing elliptical regions of interest in the midbrain and pontine basis in the midsagittal slice, and obtaining the maximal measurement perpendicular to the major axis of the ellipse

^d^As obtained at the mid-mammillary-body level

**Table 4:** Diagnostic accuracy of quantitative structural MR-based techniques including DWI, MTI, iron-sensitive sequences and NMI for the diagnosis of APD

References	Cohort size	Magnetic field	Main results	Discriminator	Accuracy, %
Diffusion imaging^a^					
Schocke et al. (2002)	MSA-P 10/PD 11/HC 7	1.5 T	↑ Putaminal diffusivity values in MSA-P compared to PD and HC No significant group differences of diffusivity values in the other ROIs. Significant correlation between UPDRS III and putaminal diffusivity values	Putaminal diffusivity	Se 100 (MSA-P) Sp 100 (vs. PD and HC)
Seppi et al. (2003)^b^	MSA-P 12/PD 13/PSP 10	1.5 T	↑ Diffusivity values in putamen, globus pallidus, and caudate nucleus in PSP compared to PD No differences of diffusivity values of the different ROIs between PSP and MSA-P	Putaminal diffusivity	Se 100 (MSA-P) Sp 100 (vs. PD) Se 90 (PSP) Sp 100 (vs. PD)
Seppi et al. (2004)^c^	MSA-P 15/PD 17/HC 10	1.5 T	↓ S/FC ratios and higher striatal diffusivity values in MSA-P compared to both PD and HC No significant differences in S/FC ratios and striatal diffusivity values between PD and HC Higher overall predictive accuracy of striatal diffusivity values (97%) compared to IBZM S/FC ratio (75%)	Striatal diffusivity	Se 93 (MSA-P) Sp 100 (vs. PD and HC)
Schocke et al. (2004)	MSA-P 11/PD 17/HC 10	1.5 T	↑ Putaminal and pallidal diffusivity values in MSA-P compared to both PD and HC Complete discrimination between MSA-P vs. PD and HC with putaminal diffusivity values in *y*- and *z-* direction Significant correlation between UPDRS III and putaminal diffusivity values	Putaminal diffusivity	Se 100 (MSA-P) Sp 100 (vs. PD and HC)
Shiga et al. (2005)	MSA 11 (8 MSA-C, 3 MSA-C)/HC 10	1.5 T	↓ FA values in MCP, basis pontis and internal capsule Significant negative correlation of MCP FA values with ataxia scores	FA in MCP	Se 100 (MSA-P and MSA-C) Sp 100 (vs. HC)
Seppi et al. (2006a)	MSA-P 15/PD 20/HC 11	1.5 T	↑ Diffusivity values in the entire, anterior, and posterior putamen in MSA-P compared to PD and HC ↑ Diffusivity values in the posterior compared to the anterior putamen in MSA-P No significant differences between posterior and anterior putamen in PD and HC	Putaminal diffusivity	Se 93 (MSA-P) Sp 100 (vs. PD and HC)
Seppi et al. (2006b)	MSA-P 15/PD 20/HC 11	1.5 T	↑ Diffusivity values in the entire, anterior, and posterior putamen in MSA-P compared to PD and HC ↑ Diffusivity values in the posterior compared to the anterior putamen in MSA-P No significant differences between posterior and anterior putamen in PD and HC	Posterior putaminal diffusivity	Se 100 (MSA-P) Sp 100 (vs. PD and HC)
Nicoletti et al. (2006b)	MSA-P 16/PD 16/PSP 16/HC 15	1.5 T	↑ Putaminal diffusivity values in MSA-P compared to PD and HC ↑ MCP diffusivity values in MSA-P compared to PD, HC and PSP	MCP diffusivity	Se 100 (MSA-P) Sp 100 (vs. all groups)
				Putaminal diffusivity	Se 100 (MSA-P) Sp 100 (vs. PD and HC), 81 (vs. PSP)
Kollensperger et al. (2007)	MSA-P 9/PD 9/HC 16	1.5 T	↑ Putaminal diffusivity values in MSA-P compared to PD and HC No significant differences of blood pressure response to passive tilt between PD and MSA-P DWI was superior to both tilt table testing and MIBG scintigraphy in the differential diagnosis of MSA-P vs. the other groups	Putaminal diffusivity	Se 100 (MSA-P) Sp 100 (vs. all groups)
Paviour et al. (2007)	MSA-P 11/PD 12/PSP 20/HC 7	1.5 T	↑ Diffusivity values in the MCP and rostral pons in MSA-P compared to PSP and PD Significant correlation between diffusivity values in rostral pons and H&Y in MSA-P Significant correlation between globus pallidum diffusivity values and H&Y and UPDRS II and III	MCP diffusivity	Se 91 (MSA-P) Sp 82 (vs. all groups), 84 (vs. PSP)
Ito et al. (2007)	MSA 20 (MSA-P 10, MSA-C 10)/PD 21/HC 20	3.0 T	↑ Diffusivity values and significant lower FA values in the pons, cerebellum, and putamen in MSA compared to PD and HC All patients that had both significant low FA and high diffusivity values in each of the three regions were MSA-P cases, and those that had both normal FA and diffusivity values in the pons were all PD cases	Diffusivity pons	Se 70 (MSA-P) Sp 70 (vs. PD)
				Diffusivity cerebellum	60 (MSA-P) Sp 88 (vs. PD)
				Diffusivity putamen	70 (MSA-P) Sp 64 (vs. PD)
				FA pons	70 (MSA-P) Sp 100 (vs. PD)
				FA cerebellum	70 (MSA-P) Sp 64 (vs. PD)
				FA putamen	70 (MSA-P) Sp 88 (vs. PD)
				Both low FA and high Diffusivity values in any of the three areas	90 (MSA-P) Sp 100 (vs. PD)
Nicoletti et al. (2008)	MSA-P 15/PD 16/PSP 28/HC 15	1.5 T	↑ SCP diffusivity values in PSP compared to MSA-P, PD, and HC Assessment of diffusivity values in the SCP was not possible in two patients due to methodological reasons	SCP diffusivity	Se 100 (PSP) Sp 100 (vs. PD and HC) Se 97 (PSP) 93 (vs. MSA-P)
Rizzo et al. (2008)	PSP-RS 10/CBS 7/PD 13/HC 9	1.5 T	↑ Diffusivity in putamen and SCP in PSP-RS vs. PD ↑ Diffusivity in putamen in CBS vs. PD ↑ Hemispheric MD in CBS vs. PD and PSP-RS ↓ Hemispheric symmetry ratio in CBS vs. PD and PSP-RS	Putaminal diffusivity	Se 80 (PSP-RS) Sp 77 (vs. PD) Se 86 (CBS) Sp 92 (vs. PD)
				SCP diffusivity	Se 90 (PSP-RS) Sp 85 (vs. PD)
				Hemispheric MD	Se 86 (CBS vs. PD) Sp 85 (CBS vs. PD) Se 100 (CBS vs. PSP-RS) Sp 90 (CBS vs. PSP-RS)
				Hemispheric symmetry ratio	Se 100 (CBS vs. PD and PSP-RS) Sp 100 (CBS vs. PD and PSP-RS)
Chung et al. (2009)	PD 12/MSA-P 10/HC 10	1.5 T	↑ Diffusivity in dorsal putamen and MCP in MSA-P vs. PD and HC	Diffusivity dorsal putamen	Se 67 (MSA-P) Sp 80 (vs. PD)
				Diffusivity MCP	Se 92 (MSA-P) Sp 100 (vs. PD)
Boelmans et al. (2010)	CBS 14/PD 14/HC 14	1.5 T	↑ MD in corpus callosum in CBS vs. PD and HC ↓ FA in middle-dorsal (sensory) corpus callosum in CBS vs. PD and HC No differences between PD and HC	MD in corpus callosum	Se 79 (CBS) Sp 79 (vs. PD)
				MD in middle-dorsal corpus callosum	Se 86 (CBS) Sp 71 (CBS vs. PD)
Wang et al. (2012a, b)	MSA 31 (MSA-P 12, MSA-C 19)/PD 20/HC 20	1.5 T	↑ Diffusivity values in the MCP and cerebellum in MSA-P and MSA-C compared to HC ↓ FA values in the pyramidal tract, MCP, and white matter of the cerebellum in MSA-C and MSA-P compared to HC No significant diffusivity changes in PD compared to HC	Diffusivitycerebellum	Se 95 (MSA-C), 75 (MSA-P) Sp 85 (vs. PD and HC)
				FA cerebellum	Se 95 (MSA-C), 83 (MSA-P) Sp 80 (vs. PD and HC)
				Diffusivity cerebellum	Se 58 (MSA-C) Sp 100 (vs. PD and HC)
				Diffusivity basal ganglia	Se 52 (MSA) Sp 90 (vs. HC)
Nicoletti et al. (2013)	9 MSA-P/7 MSA-C/17 PSP-RS/10 PD/10 HC	1.5 T	↑ Median MD values in whole infratentorial compartment, brainstem and cerebellum in MSA-P and MSA-C vs. other groups ↑ MD values in the cerebellar vermis in MSA-C vs. MSA-P ↑ MD in the vermis in PSP vs. PD and HC	MD in whole infratentorial compartment	Se 100 (MSA-C and MSA-P) Sp 100 (MSA-C and MSA-P vs. other groups)
				MD in the vermis	Se 100 (PSP vs. HC and PD) Sp 100 (PSP vs. HC and PD)
Umemura et al. (2013)	MSA 20/PD 118	1.5 T	↑ Putaminal diffusivity in MSA-P vs. PD	Putaminal diffusivity	Se 85 (MSA-P) Sp 89 (vs. PD)
Surova et al. (2013)	PD 10/MSA-P 12/PSP 16/HC 16	3.0 T	↑ MD, RD, and ↓ FA in corpus callosum in PSP vs. PD and HC Increased apparent area coefficient in frontal and parietal cingulum and ↑ MD in corticospinal tract in PSP vs. PD ↑ RD in MSA-P vs. PD	Apparent area coefficient in frontal and parietal cingulum	Se 87 (PSP) Sp 80 (PSP vs. PD) AUC = 0.88
				MD in corticospinal tract	Se 94 (PSP) 80 (PSP vs. PD) AUC = 0.85
				MD in corpus callosum	Se 81 (PSP) 80 (PSP vs. PD) AUC = 0.85
Prodoehl et al. (2013)	PD 15/MSA 14/PSP 12/ ET 14/HC 17	3.0 T	Multi-target imaging approach focused on the basal ganglia and cerebellum accurately classifies control subjects and patients with PD, MSA-P, PSP, and ET SN, putamen, caudate, and MCP were the most frequently selected brain regions across classifications	Model using DTI measures from the putamen, pallidum, SN, red nucleus, and MCP	Se 92 (HC) Sp 91 (HC vs. PD, MSA, PSP) AUC = 0.99
				Model using DTI measures from the putamen, SN, and dentate nucleus	Se 90 (PD) Sp 100 (PD vs. MSA, PSP) AUC = 0.99
				Model using DTI measures from the SN and MCP	Se 94 (PD) Sp 100 (PD vs. MSA) AUC = 0.99
				Model using DTI measures from the putamen and SN	Se 87 (PD) Sp 100 (PD vs. PSP) AUC = 0.96
				Model using DTI measures from caudate and MCP	Se 90 (MSA) 100 (MSA vs. PSP) AUC = 0.97
Baudrexel et al. (2014)	PD 13/PSP 8/MSA-P 11/HC 6	3.0 T	↑ MD in posterior putamen in MSA-P vs. PD, PSP, and HC ↑ MD in anterior putamen in MSA-P vs. PD	MD posterior putamen	Se 73 (MSA-P) Sp 100 (vs. other groups) AUC = 0.89
Meijer et al. (2015a, b)	Clinically uncertain parkinsonism 60 (of which *n* = 30 developed PD and *n* = 19 patients developed atypical parkinsonism: 12 MSA-P/3 PSP/3 DLB/1 CBS)	3.0 T	DTI did not significantly improve the diagnostic accuracy of conventional brain MRI to differentiate the group of AP from PD The diagnostic accuracy to identify MSA-P was slightly increased by combining conventional MRI with DTI ↑ MD of the centrum semiovale, body corpus callosum, putamen, external capsule, midbrain, superior cerebellum, and SCP was found in clinically uncertain parkinsonism that developed AP ↑ MD of the putamen in clinically uncertain parkinsonism that developed MSA-P vs. PD ↑ MD in the midbrain and SCP in clinically uncertain parkinsonism that developed PSP vs. PD and MSA-P	MD in putamen, midbrain, and SCP	AUC = 0.75 (atypical parkinsonism vs. PD)
				Combination of conventional brain MRI and DTI	AUC = 0.83 (atypical parkinsonism vs. PD) AUC = 0.85 (MSA vs. other group)
Surova et al. (2015)	PSP 27/MSA-P 11/PD 10/HC 21	3.0 T	↑ MD in thalamus, ventral anterior, and ventral posterior thalamic nuclei and midbrain in PSP vs. MSA-P, PD, and HC ↑ MD in pons and putamen in MSA-P vs. PD and HC ↑ MD and decreased FA of bilateral DRTT in PSP vs. MSA-P, PD, and HC ↓ Thalamus, putamen, and pallidus volumes and midbrain area in PSP vs. MSA-P, PD, and HC in both cohorts ↓ Putamen and pallidus volumes in MSA-P vs. PD and HC	MD of the thalamus	Se 81 (PSP) Sp 77 (vs. PD and MSA-P) AUC = 0.81 (vs. PD and MSA-P
				MD right DRTT	Se 92 (PSP) Sp 81 (vs. PD and MSA-P), AUC = 0.94 (vs. PD and MSA-P
				MD midbrain	Se 81 (PSP) Sp 81 (vs. PD and MSA-P) AUC = 0.90 (vs. PD and MSA-P
Planetta et al. (2016)	PD 18/MSA 18/PSP 18/HC 18	3.0 T	↑ FW in the anterior and posterior SN of PD, MSA, and PSP vs. HC ↑ free-water in all regions except the dentate nucleus, subthalamic nucleus, and corpus callosum of MSA, and in all regions examined for PSP ↑ FW-corrected FA values for MSA in the putamen and caudate compared with PD and HC ↑ FW-corrected FA values for PSP in the putamen, caudate, thalamus, and vermis, and decreased in the SCP and corpus callosum compared with PD and HC	Model including FW in posterior SN and FA in SCP	Se 94 (HC) Sp 83 (HC vs. all groups) AUC 0.93
				Model including FW in SCP and FA in SCP putamen, vermis, and corpus callosum	Se 93 (PD vs. MSA, PSP) Sp 93 (PD vs. MSA, PSP) AUC = 0.94
				Model including FW in thalamus and cerebellar lobule V and FA in caudate nucleus	Se 95 (PD vs. MSA) Sp 89 (PD vs. MSA) AUC = 0.97
				FW in SCP	Se 100 (PD vs. PSP) Sp 100 (PD vs. PSP) AUC = 1.00
				Model including FW in pedunculopontine nucleus and subthalamic nucleus	Se 95 (MSA) Sp 95 (MSA vs. PSP) AUC = 0.97
Sako et al. (2016)	MSA-P 11/PD 36	1.5 and 3.0 T	Better AUC for MCP width and putaminal diffusivity Similar AUCs were seen in all patients with different disease duration and with different field strengths (1.5 or 3.0 T)	MCP width	AUC = 0.93 (MSA-P vs. PD)
				Putaminal diffusivity	AUC = 0.83 (MSA-P vs. PD)
				Cerebellar diffusivity	AUC = 0.73 (MSA-P vs. PD)
Magnetization transfer imaging					
Eckert et al. (2004)	MSA 12/PD 15/PSP 10/HC 20	1.5 T	Change in the MTR in the globus pallidus, putamen, caudate nucleus, SN Stepwise linear discriminant analysis provided a good classification of the individual patients into the different disease groups	Model including MTRs of globus pallidus, putamen and caudate nucleus (using stepwise linear discrimination model)	Overall correct classification 75% 95% of APD and 100% of non-APD classified correctly 75% of PD, 80% of HC, 58% of MSA and 90% of PSP classified correctly
Iron-sensitive sequences					
von Lewinski et al. (2007)	MSA 52/PD 88/HC 29	1.0 T	Signal loss of the dorsolateral putamen on T2* GE sequences in MSA Hyperintense lateral putaminal rim on FLAIR sequences MSA	T2* Signal loss dorsolateral putamen	Se 69 (MSA) Sp 91 (vs. PD and HC)
				T2* Hyperintense putaminal rim	Se 48 (MSA) Sp 93 (vs. PD and HC)
				T2* Signal loss dorsolateral putamen and hyperintense putaminal rim	Se 42 (MSA) Sp 97 (vs. PD and HC)
				SI _PUT/CAUD_	Se 65 (MSA) Sp 95 (vs. PD)
Gupta et al. (2010)	MSA-P 12/PSP 12/PD 11	1.5 T	↑ Red nucleus hypointensity in PSP compared to MSA-P and PD using SWI ↑ Putaminal hypointensity in PSP compared to PD using SWI No significant differences in putaminal hypointensity between PSP and MSA-P or MSA-P and PD using SWI	SWI hypointensity score >2 (red nucleus)	Se 67 (PSP) Sp 82 (vs. PD) 83 (vs. MSA-P)
Arabia et al. (2010)	MSA 20/PSP 41/PD 189/HC 150	1.5 T	↑ Frequencies of putaminal hypointensities in MSA-P and PSP compared to PD and HC using T2* GE sequences with 15 ms time echo	Putaminal hypointensities^d^	Se 55 (MSA), 25 (PD), 44 (PSP) Sp 93 (vs. HC)
Sakurai et al. (2010)	MSA-P 10/PD 14/HC 10	1.5 T	↑ Grade of putaminal hypointensity in MSA-P compared to PD and HC on all 3D-PRESTO, T2*, and T2 sequences Significant differences in the mean grade of putaminal hypointensity in MSA-P among 3D-PRESTO, T2*, and T2 sequences	Putaminal hypointensity (3D PRESTO)^d^	Se 90 (MSA-P) Sp 79 (vs. PD), 70 (vs. HC)
				Putaminal atrophy (3D PRESTO)^d^	Se 70 (MSA-P) Sp 100 (vs. PD and HC)
Wang et al. (2012a, b)	PD 16/MSA-P 8/HC 44	1.5 T	↑ Iron content in putamen and thalamus in MSA-P vs. PD High-iron-deposition-percentage area provides slightly better accuracy than mean shift values	High-iron-deposition-percentage area in putamen	AUC = 0.88 (MSA-P vs. PD)
				High-iron-deposition-percentage area in pulvinar thalamus	AUC = 0.79 (MSA-P vs. PD)
Han et al. (2013)	PSP 11/MSA-P 12/HC 20	3.0 T	↑ Iron deposition in PSP and MSA-P vs. HC and PD ↑ Iron concentration of the red nucleus, SN, globus pallidus and thalamus in PSP vs. MSA-p ↑ Putaminal iron concentration in MSA vs. PSP ↑ Iron-related hypointense signals in the posterolateral putamen and adjacent lateral aspect of the globus pallidus in MSA-P ↑ Hypointense signals in the anterior and medial aspects of the globus pallidus and thalamus in PSP	Putaminal mean phase shift values	AUC = 0.84 (MSA vs. PD and PSP)
				Mean phase shift values in globus pallidus	AUC = 0.87 (PSP vs. PD and MSA)
				Mean phase shift values in thalamus	AUC = 0.88 (PSP vs. PD and MSA)
Yoon et al. (2015)	PD 30/MSA-P 17	3.0 T	↓ Signal intensity of bilateral posterior halves, mean values of the anterior and posterior halves, and the dominant-side posterior half of the putamen in MSA-P vs. PD	Signal intensity of the posterior part of putamen	AUC = 0.95 (MSA vs. PD)
Multimodal imaging					
Barbagallo et al. (2016)	PD 26/MSA 29 (MSA-P 16, MSA-C 13).	3.0 T	Volume loss and both higher mean diffusivity values and T2* relaxation rates values in their putamina as well as higher caudate mean diffusivity values in MSA vs. PD No nigral changes between groups	Combination of T2* relaxation rates values and MD in putamen	AUC = 0.96 (PD vs. MSA-P)

*APD * atypical parkinsonian disorders, Se sensitivity, *Sp* specificity, *AUC* area under the curve, ↑ significant higher, ↓ significant lower, *MSA* multiple system atrophy, *MSA-P* parkinsonian variant of MSA, *MSA-C* cerebellar variant of MSA, *PD* Parkinson’s disease, *PSP* progressive supranuclear palsy, *PSP-RS* progressive supranuclear palsy-Richardson Syndrome, *HC* healthy controls, *CBS* corticobasal syndrome, *MRI* magnetic resonance imaging, *T* tesla, *MCP* middle cerebellar peduncle, *SCP* superior cerebellar peduncle, *SN* substantia nigra, *DRTT* dentatorubrothalamic tract, *GE* gradient echo, *FLAIR* fluid-attenuated inversion recovery, *AUC* area under the curve, *ROI* region of interest, *DWI* diffusion-weighted imaging, *DTI* diffusion tensor imaging, *SWI* susceptibility-weighted imaging, *S/FC* activity ratios of striatal to frontal cortex uptake, *IBZM* [123I]benzamide-SPECT imaging, *HSR* hemispheric symmetry ratio, *ADC* apparent diffusion coefficient, *MD* mean diffusivity, *FA* fractional anisotropy, *FW* free water, *MTI* magnetization transfer imaging, *MTR* magnetization transfer ratio, *SI*_*PUT/CAUD*_ signal intensity dorsolateral putamen/signal intensity head of caudate nucleus

^a^In the studies by Schocke et al. (2002) and Seppi et al. (2003, 2004), ADC was measured in *z*-slice direction only; in the other studies ADCs were averaged (ADCave) over three orthogonal measurements, thus representing the Trace (D) or diffusivity

^b^Including all patients studied by Schocke et al. (2002)

^c^Including all patients studied by Seppi et al. (2003)

^d^Sensitivity and specificity refer to the qualitative inspection of iron-sensitive images

### Quantitative assessment of regional cerebral atrophy

As an indirect method of measuring regional brain atrophy, groups have applied simple quantitative measures of diameters, areas and volumes including ROI-based assessment of various structures on MRI for differential diagnostic purposes (see Table 3) (Hotter et al. 2009; Mahlknecht et al. 2010). In terms of infratentorial atrophy, several studies have demonstrated that MSA is associated with a relatively greater pontine and MCP atrophy compared to PSP and PD, whereas patients with PSP have a relatively greater midbrain and SCP atrophy compared to MSA and PD (Warmuth-Metz et al. 2001; Righini et al. 2004; Oba et al. 2005; Paviour et al. 2005; Nicoletti et al. 2006a; Quattrone et al. 2008; Hotter et al. 2009; Gama et al. 2010).

As an indirect sign of midbrain atrophy in PSP, the anteroposterior midbrain diameter has been described to be reduced in PSP compared to non-PSP parkinsonism, however, with overlapping individual results (Warmuth-Metz et al. 2001; Savoiardo 2003). More recently, new approaches assessing the midbrain diameter have been introduced (Massey et al. 2013; Kim et al. 2015). By placing elliptical ROIs in the midbrain and pontine basis in the midsagittal slice, the maximal measurement perpendicular to the major axis of the ellipse has been obtained. Patients with PSP could be separated from patients with MSA and PD with a high diagnostic accuracy either using the midbrain diameter or the midbrain to pons diameter ratio (m_d_/p_d_-ratio) (Massey et al. 2013). Interestingly, this method was validated in a post-mortem cohort of patients with neurodegenerative parkinsonism (Massey et al. 2013). Another study used a different approach in obtaining the midbrain diameter by measuring the length from the interpeduncular fossa to the center of the cerebral aqueduct at the mid-mammillary-body level, adjusted according to the anterior commissure–posterior commissure length in patients with PD and PSP by comparing this measure to the MR parkinsonism index (MRPI) and the midbrain to pontine area ratio (m_a_/p_a_-ratio) (Kim et al. 2015). The midbrain diameter as obtained at the mid-mammillary-body level discriminated PSP from PD with an area under the curve (AUC) of 0.76 which was similar to the discriminatory power of the m_a_/p_a_-ratio and significant better to the MRPI (AUC of 0.69). These new approaches in assessing the midbrain diameter provided good discriminatory power, but confirmative studies are warranted.

Midsagittal measurements of brainstem areas reveal decreased midbrain areas in PSP patients compared to non-PSP parkinsonian patients and decreased pontine areas in MSA patients compared to non-MSA parkinsonian patients (Hotter et al. 2009). As single measurements of these structures have been shown not to adequately distinguish between neurodegenerative parkinsonian disorders, especially MSA and PSP, the ratio between m_a_/p_a_-ratio was found to be significantly smaller in patients with PSP compared to other groups and to differentiate better than the single measurement (Oba et al. 2005; Cosottini et al. 2007).

Moreover, it has been shown that the MRPI, which is the product of the ratio of pons area to midbrain area in midsagittal expanse multiplied by the ratio of the width of MCPs and SCPs ([area pons/area midbrain] × [width MCP/width SCP]), is able to differentiate PSP patients from non-PSP parkinsonism including PD and MSA as well as healthy controls (Quattrone et al. 2008; Hussl et al. 2010; Morelli et al. 2011a, b; Zanigni et al. 2016). Compared with m_a_/p_a_-ratio, the MRPI seems to better differentiate PSP from MSA-P, while the m_a_/p_a_-ratio is a better discriminator between PSP and PD (Hussl et al. 2010). Both a decreased m_a_/p_a_-ratio as well as an increased MRPI seem to distinguish PSP from MSA, PD, and healthy controls; however, there are some overlapping individual values (Oba et al. 2005; Quattrone et al. 2008; Hussl et al. 2010; Longoni et al. 2011; Morelli et al. 2011a, b). However, a large multicentre retrospective study of 391 patients with established neurodegenerative parkinsonism, including 106 patients with PSP, favours the midsagittal m_a_ and the m_a_/p_a_ ratio to differentiate PSP from MSA and PD instead of using the MRPI (Moller et al. 2017). By contrast to earlier studies (Oba et al. 2005; Hussl et al. 2010; Morelli et al. 2011a) where cutoff values for the MR planimetric measurements were given for PSP vs. non-PSP parkinsonism, cutoff values for the MR planimetric measurements in this study (Moller et al. 2017) were calculated separately for PSP vs. each other parkinsonian group. For clinical and research (e.g. early detection of PSP for treatment studies) purposes, however, the clinician or researcher intends to identify PSP among a group of patients with (degenerative) parkinsonism. Moreover, one study in patients with clinically uncertain parkinsonism has suggested that an abnormal MRPI may predict PSP with a diagnostic accuracy of 93% (Morelli et al. 2011a, b). However, the patients with clinically uncertain parkinsonism in this study were in advanced disease stages presenting with atypical signs (falls within the first year, slowness of vertical saccades, freezing within the first 3 years of disease), and the inclusion criteria applied in the study suggested high probability for involving patients with atypical parkinsonism. We studied the MRPI in 81 patients who were clinically uncertain parkinsonism (including 15 patients who developed PSP and 11 patients who developed MSA) due to their early disease stage and found that an abnormal MRPI predicts PSP with a diagnostic accuracy of 85% (unpublished data). Very recently, an automated method for the MRPI calculation has been established and validated in a large cohort of 88 patients with clinically established PSP, 234 PD patients and 117 controls showing a diagnostic accuracy of 95% in separating PSP from PD (Nigro et al. 2016).

To characterize regional cerebral volume differences in patients with neurodegenerative parkinsonian disorders, manual or semi-automated ROI-techniques have been used. Indeed, volume loss of different supratentorial and infratentorial brain structures measured by MR volumetry with semi-automatic segmentation techniques on an ROI approach has been reported in patients with APDs, but differentiation between neurodegenerative parkinsonian disorders using individual structure volumetry is limited due to overlapping individual values (Schulz et al. 1999; Cordato et al. 2002; Groschel et al. 2004; Paviour et al. 2006b; Seppi and Poewe 2010; Lee et al. 2013b). Using MRI-based fully automated segmentation software (FreeSurfer), a more recent study evaluated 72 patients with PD, 15 with MSA-P, 32 with PSP, and 46 control subjects, assessing several cerebral and subcortical regions (Messina et al. 2011). No volumetric differences were found between PD and controls, while volumes of the cerebellum, putamen, pallidum, hippocampus, and brainstem were significantly reduced in MSA-P and PSP compared to patients with PD and controls. PSP and MSA-P patients only differed in thalamic volumes, which were significant smaller in the PSP group compared to the other groups (Messina et al. 2011). In this study, no diagnostic accuracy values were given.

A plethora of studies using VBM have been performed in patients with atypical parkinsonism showing not only basal ganglia and infratentorial volume loss confirming ROI-based volumetric studies but also volume loss in several mainly frontal cortical regions in patients with atypical parkinsonism (Brenneis et al. 2004, 2007; Price et al. 2004; Cordato et al. 2005; Boxer et al. 2006; Padovani et al. 2006; Chang et al. 2009; Agosta et al. 2010; Tzarouchi et al. 2010; Lee et al. 2011; Takahashi et al. 2011; Ghosh et al. 2012; Giordano et al. 2013; Lagarde et al. 2013; Shigemoto et al. 2013; Whitwell et al. 2013; Yu et al. 2015; Fiorenzato et al. 2017). Very recently, two meta-analyses of VBM studies in patients with atypical parkinsonism have been performed (Shao et al. 2015; Yu et al. 2015). One of these studies analysed patients with MSA-P including 72 patients with MSA-P from 5 studies, 643 controls from 28 studies and 639 patients with PD from 23 patients (Shao et al. 2015). Interestingly, for patients with a disease duration up to 5 years, compared with PD, the decrease in grey matter (GM) volume focused on the bilateral putamen and claustrum in MSA-P, while for patients with a disease duration up to 3 years, no significant GM volume difference was found between MSA-P and PD suggesting that the atrophy of bilateral putamen or claustrum is not a neuroanatomical marker for distinguishing MSA-P from PD during the early stage by using VBM (Shao et al. 2015). A second meta-analysis included 404 patients with PD, 87 with MSA-P, 165 patients presenting with a corticobasal syndrome (CBS) (including also patients with CBD), and 176 with PSP from 39 published VBM articles (Yu et al. 2015). This VBM meta-analysis identified distinctive patterns of GM volume reduction in CBD, PSP and MSA-P with regions of atrophy distinctive to each disease, including the left parietal lobe in CBD, thalamus and insula in PSP, and putamen in MSA-P, while mild overlap in GM atrophy was found between CBD and PSP, as well as PSP and MSA-P (Yu et al. 2015).

Despite many advantages of voxel-based analysis, including its independence from operators due to automated detection, at this time it is not appropriate for routine diagnostic work-up of individual patients since it involves group-wise comparisons (Mahlknecht et al. 2010). Furthermore, in performing a voxel-based study many methodological options are available and known for pitfalls which are summarized in a comprehensive review (Ridgway et al. 2008).

Intriguingly, a novel approach for automated differentiation of parkinsonian syndromes on single-patient level using a fully automated method for quantitative MRI analysis using atlas-based voxel-based volumetry combined with SVM classification has been introduced in a study including 73 healthy controls, 204 patients with PD, 106 patients with PSP and 81 patients with MSA (60 of them with the MSA-P subtype) (Huppertz et al. 2016). Compared with the healthy control group, the largest atrophy for the PSP groups was found in the midbrain (−15%), midsagittal midbrain tegmentum plane (−20%), and superior cerebellar peduncles (−13%), and for the MSA-P group in the putamen (−23%) yielding the majority of binary SVM classifications between the groups resulted in balanced diagnostic accuracies of 80% and more. Volume changes of midbrain, basal ganglia, and cerebellar peduncles had the largest relevance for classification in this study (Huppertz et al. 2016). Another approach of automated differentiation of parkinsonian syndromes on single-patient level has been performed using automated subcortical volume segmentation with the MRI-based software tool FreeSurfer followed by a machine-learning method-derived classification algorithm (i.e. C4.5 decision tree algorithm) (Scherfler et al. 2016). In this study, the decision algorithm built by including 22 segmented subcortical regions was applied to 40 patients with PD, 40 with MSA-P and 30 with PSP in early to moderately advanced stages. The midbrain and putaminal volume as well as the cerebellar grey matter compartment were identified as the most significant brain regions to construct a prediction model. Contrary to the former report (Huppertz et al. 2016), the study population in this study was separated into a validation and a test cohort in order to strengthen the results. The diagnostic accuracy for PD vs. MSA or PSP was 97%, which was in contrast to the diagnostic accuracy of 63% based on validated clinical consensus criteria at the time of MRI acquisition suggesting that automated volume segmentation of subcortical brain areas improves diagnostic accuracy in patients presenting with early to moderately advanced stage parkinsonism (Scherfler et al. 2016).

### Quantitative structural MR-based techniques

#### Diffusion imaging

Over the past 15 years, there has been growing interest in the use of diffusion imaging for the differential diagnosis of atypical parkinsonism from PD. Several studies performed on an ROI basis found that diffusion imaging discriminates MSA-P (even in early disease stages) from PD as well as healthy subjects on the basis of putaminal diffusivity measures values (see Table 4) (Schocke et al. 2002, 2004; Seppi et al. 2003, 2004, 2006a, b; Nicoletti et al. 2006b; Ito et al. 2007; Kollensperger et al. 2007; Chung et al. 2009; Meijer et al. 2013, 2015b; Umemura et al. 2013; Baudrexel et al. 2014; Barbagallo et al. 2016). Few studies compared the diagnostic value of putaminal diffusivity to either dopamine D2 receptor binding with [132-I]-iodobenzamide-single-photon emission computed tomography (IBZM-SPECT) (Seppi et al. 2004), cardiac [132-I]-meta-iodobenzylguanidine (MIBG) uptake (Kollensperger et al. 2007; Sako et al. 2016) or [18-F]-fluorodeoxyglucose positron emission tomography (FDG-PET) (Baudrexel et al. 2014). Putaminal diffusivity measures were more accurate compared with IBZM-SPECT, cardiac MIBG and FDG-PET. In line with the known underlying neuropathology in MSA-P, a more severe involvement of posterior compared with anterior putaminal diffusivity was found in patients with MSA-P (Seppi et al. 2006b; Pellecchia et al. 2009). Moreover, PD subjects with longer disease duration and concomitant white matter changes (WMC) might also have increased putaminal diffusivity (Esterhammer et al. 2015). Eventhough most studies found an increased putaminal diffusivity in MSA-P compared with PD at 1.5 T (Schocke et al. 2002, 2004; Nicoletti et al. 2006b; Seppi et al. 2006a, b; Ito et al. 2007; Pellecchia et al. 2009; Meijer et al. 2013, 2015b; Baudrexel et al. 2014; Barbagallo et al. 2016), two studies did not confirm this finding (Paviour et al. 2007; Wadia et al. 2013), presumably due to longer PD disease duration in these two studies compared to other cohorts (Seppi et al. 2003; Blain et al. 2006; Nicoletti et al. 2006a, b; Ito et al. 2007).

Abnormal diffusion metrics in the MCP have been reported for MSA (Kanazawa et al. 2004; Shiga et al. 2005; Blain et al. 2006; Nicoletti et al. 2006a, b; Paviour et al. 2007; Chung et al. 2009; Pellecchia et al. 2009), as well as abnormal diffusion metrics in the SCP for PSP (Blain et al. 2006; Nicoletti et al. 2006b; Rizzo et al. 2008), with most of these studies reporting good discrimination between MSA and PSP as well as from PD and healthy controls, respectively (Nicoletti et al. 2006b; Pellecchia et al. 2009; Rizzo et al. 2008). However, while diffusivity in the MCP has been reported to have a high diagnostic accuracy for MSA-P in some publications (Nicoletti et al. 2006b; Paviour et al. 2007), this could not be confirmed by others (Blain et al. 2006; Pellecchia et al. 2009). Increased putaminal diffusivity has also been reported for patients with PSP (Seppi et al. 2003; Nicoletti et al. 2006b; Rizzo et al. 2008), although discriminatory power from PD seems to be less compared to patients with MSA-P. Moreover, because putaminal diffusivity overlapped in MSA-P and PSP patients (Seppi et al. 2003; Nicoletti et al. 2006b), discrimination between these two APDs is not possible.

There are few 3.0 T diffusion imaging studies and the results are inconsistent, possibly due to increased SNRs, increased magnetic susceptibility effects, and increased echo-planar image distortion at 3.0 T that may affect diffusion imaging findings compared to 1.5 T (Seppi et al. 2003; Schocke et al. 2004; Ito et al. 2007; Focke et al. 2011a; Tsukamoto et al. 2012). Diffusivity values in the pons, cerebellum and putamen at 3.0 T were found to be significantly higher and FA values lower in MSA than in PD or controls (Ito et al. 2007 ). In differentiating MSA-P from PD using FA and diffusivity values, there was similar sensitivity (70%) and higher specificity (100%) in the pons than in the putamen and cerebellum. Another study found a significant increase of diffusivity in the globus pallidus and SN bilaterally in PSP patients vs. PD patients and controls. Furthermore, diffusivity values in the SN were higher in the PSP group compared to patients with MSA-P, and diffusion imaging showed no significant predictive power in patients with MSA-P. However, by contrast to all other reports, the authors of this study used a stimulated echo acquisition mode (STEAM)-based diffusion imaging (Focke et al. 2011a) compared to the conventional echo-planar imaging (EPI)-based diffusion imaging sequency used in the other publications. A further diffusion imaging study at 3.0 T including 25 patients with MSA, 20 with PSP, and 17 with PD as well as 18 healthy controls revealed significantly elevated diffusivity in the posterior putamen, midbrain, pons, MCP, and cerebellar white matter for the MSA group and in the globus pallidus and midbrain for the PSP group, which is in line with the characteristic lesions in MSA and PSP. Diagnostic accuracy, however, was not given in this study (Tsukamoto et al. 2012). Moreover, significantly increased diffusivity of the putamen was found in MSA-P and increased diffusivity in the midbrain and SCP in PSP compared to PD in a study including 60 parkinsonian patients presenting with clinically uncertain parkinsonism and a disease duration of less than 3 years, of whom probable diagnoses could be made in 49 patients [PD in 30, dementia with Lewy bodies (DLB) in 3, MSA-P in 12, PSP or CBS in 4] (Meijer et al. 2015b). In this study, the diagnostic accuracy of brain MRI to identify atypical parkinsonism as a group was not improved by diffusivity measures in different subcortical structures, though the diagnostic accuracy to identify MSA-P was slightly increased (from an AUC of 0.82–0.85) (Meijer et al. 2015b).

Diffusion imaging and volumetric data were analysed in an interesting study comprising a derivation cohort of 30 controls and 8 patients with PSP as well as a validation cohort of 21 controls, 27 patients with PSP, 10 with PD and 11 with MSA-P with different approaches including an ROI-based approach, tract-based spatial statistic (TBSS), and tractography (Surova et al. 2015). In the derivation cohort, reduced thalamic volumes as well as increased MD in the thalamus, SCP, and the midbrain were found in the PSP group compared to controls, while in the validation cohort, the results of increased MD were replicated. Moreover, tractography of the dentatorubrothalamic tract (DRTT) showed increased MD in PSP patients from both cohorts compared to controls and in the validation cohort in PSP compared to PD and MSA patients. Using diffusion tensor tractography, the same group demonstrated disease-specific regional white matter changes in PSP, MSA and PD with the anterior portion of the corpus callosum identified as a promising region for detection of neurodegenerative changes in patients with PSP (Surova et al. 2013). This is in accordance with an earlier study, where diffusion imaging identified a PSP-associated microstructural alteration pattern in the frontal lobes and in the corpus callosum including the corresponding bilateral callosal radiation tracts (Rosskopf et al. 2014). Interestingly, abnormal DTI metrics in the corpus callosum have also been reported by an earlier 1.5 T DTI study in patients with CBS (Boelmans et al. 2010), underpinning the close relationship of the two disorders CBS and PSP. Moreover, minimally operator-dependent diffusivity histogram analyses of the whole cerebellar hemispheres have been shown in a further study to distinguish between patients with MSA from patients with PSP and PD (Nicoletti et al. 2013). Another recent study investigated a multi-target diffusion imaging approach using different DTI metrics focused on the basal ganglia and cerebellum in 15 patients with PD, 15 patients with MSA-P, 14 patients with PSP, 12 patients with ET and 17 healthy controls (Prodoehl et al. 2013). The SN, putamen, caudate, and MCP were the most frequently selected brain regions across classifications. Sensitivities and specificities of the group-wise comparisons were high (sensitivities ≥87% and specificities ≥88%) with varying brain targets for each comparison suggesting that using DTI of the basal ganglia and cerebellum accurately classifies subjects diagnosed with PD, atypical parkinsonism, and ET.

When comparing FW and FW-corrected FA maps across 72 subjects (18 healthy controls, patients with PD, MSA and PSP, each) in the basal ganglia, midbrain, thalamus, dentate nucleus, cerebellar peduncles, cerebellum, and corpus callosum, FW was increased in the anterior and posterior SN of patients with PD, MSA, and PSP vs. controls (Planetta et al. 2016). Moreover, FW was elevated in all regions examined in the patients with PSP and in all regions except the dentate nucleus, subthalamic nucleus (STN), and corpus callosum in the patients with MSA. Compared with controls, the putamen and caudate showed increased FW-corrected FA values in the MSA and PSP group, while for the PSP group FW-corrected FA values were additionally increased in the thalamus and vermis, and decreased in the SCP and corpus callosum. These data suggest that in MSA and PSP a broad network of elevated FW and altered FW-corrected FA includes the SN, basal ganglia, thalamus, and cerebellum. Interestingly, for all disease group comparisons, diagnostic accuracy was high with an SVM tenfold cross-validation AUC varying between 0.93 and 1.00 (Planetta et al. 2016).

#### Magnetization transfer imaging (MTI)

There are few studies reporting abnormalities of the basal ganglia and SN on MTI in patients with PD, MSA and PSP (Eckert et al. 2004; Anik et al. 2007; da Rocha et al. 2007). Different studies reported a decrease in MTR in the SNc (Tambasco et al. 2003; Eckert et al. 2004; Anik et al. 2007). One study investigated the potential of MTI in the differential diagnosis of neurodegenerative parkinsonism (Eckert et al. 2004). The main finding was a change in the MTRs in the globus pallidus, putamen, caudate nucleus, SN, and white matter in PD, MSA, and PSP patients, matching the pathological features of the underlying disorder. MTRs were significantly reduced in the putamen in MSA patients compared with PD patients and healthy controls as well as in the SN in patients with PSP, MSA, and PD. By application of stepwise discriminant analysis, there was a good discrimination of PD patients and controls from the MSA and PSP patients (Eckert et al. 2004).

A recent multimodal MRI study showed reduced MTR values in the putamen of patients with MSA-P; however, this finding did not allow for a differentiation between parkinsonian conditions (Focke et al. 2011a, b). Due to the limited evidence of MTI in the discrimination between PD and atypical parkinsonism, its use remains experimental.

#### Iron-sensitive MRI

Patients with atypical parkinsonism due to PSP and MSA often show putaminal changes using iron-sensitive MRI sequences to a degree that they are of significant diagnostic yield (Arabia et al. 2010; Gupta et al. 2010; Haller et al. 2012, 2013; Wadia et al. 2013; Feng et al. 2015; Meijer et al. 2015a; Yoon et al. 2015; Barbagallo et al. 2016; Sakurai et al. 2017). SWI phase images were applied for the determination of different iron-deposition patterns in several grey nuclei in 16 patients with PD, 8 patients with MSA-P and 44 age-matched healthy controls (Wang et al. 2012b). For this reason, different phase shifts as well as the high iron percentage of the area were evaluated in the entire putamen, four subregions of the putamen (upper inner region, upper outer region, lower inner region, lower outer), the pulvinar thalamus, the SN, the red nucleus, the caudate nucleus, the thalamus and the globus pallidus. The MSA-P cohort had significantly higher iron deposition in the putamen and the pulvinar part of the thalamus compared with the PD and control group, while iron deposition in the SN was similar between the MSA-P and PD group, which was significantly higher compared to controls. AUC showed higher sensitivity in differentiating MSA-P from PD by means of the high-iron-deposition-percentage area than the average phase shift. Moreover, the lower inner region of the putamen was the most valuable subregion in differentiating MSA-P from PD among the four putaminal subregions (Wang et al. 2012b).

Higher values in R2 and R2* maps within the basal ganglia in patients with MSA-P compared to patients with PD have been reported by a study using high-field MRI with 3.0 T (Focke et al. 2011a). The most marked findings, however, resulted from R2* measurements, where the best separation could be achieved in the putamen, showing that bilaterally significant R2* increases, whereas R2 mapping of the MSA-P group compared to PD showed a trend but was not statistically significant.

More recently, high-field SWI at 3.0 T was analysed by an ROI method of different brain structures in 13 controls and 65 patients presenting with clinically uncertain parkinsonism and a disease duration of less than 3 years, of whom probable diagnoses could be made in 56 patients (PD in 38, DLB and PSP in 3 each and MSA in 12) (Meijer et al. 2015a). Disease-specific scores of conventional MRI-based well-known MR abnormalities had a high specificity for atypical parkinsonism (80–90%), but sensitivity was limited (50–80%), while the presence of severe dorsal putaminal hypointensity improved the accuracy of brain MR imaging with increasing the AUC from 0.75 to 0.83 for identifying MSA-P and from 0.76 to 0.82 for identifying atypical parkinsonism as a group, respectively (Meijer et al. 2015a). Decreased putaminal hypointensity using SWI reflecting increased iron levels in MSA-P vs. PD was also reported by other reports (Lee and Baik 2011; Han et al. 2013). Decreased mean SWI signal intensities of the red and dentate nuclei were reported to occur in patient with PSP compared to PD patients in one study (Meijer et al. 2015a), while another reported decreased mean SWI signal intensities in the red nucleus and putamen. Mean phase shift values in different subcortical regions (including red nucleus, SN, caudate nucleus, globus pallidus, putamen, and thalamus) were analysed in a further study of 11 patients with PSP, 12 patients with MSA-P, 15 patients with PD, and 20 controls. Increased mean phase shift values reflecting increased iron content were found in the SN in all groups with degenerative parkinsonism, while patients with PSP and MSA-P overall demonstrated increased mean phase shift values compared to the control and PD groups. Comparing patients with PSP and MSA-P, mean phase shift values were higher in the red nucleus, SN, caudate nucleus, globus pallidus and thalamus in the PSP group, while they were higher in the putamen in the MSA group. Putaminal mean phase shift values best discriminated MSA-P from PD and PSP with an AUC of 0.84, and pallidal as well thalamic mean phase shift values best discriminated PSP from PD and MSA-P with AUCs of 0.87 and 0.88, respectively. Overall pathological iron accumulations were more prevalent and severe in PSP compared to MSA-P in this study as confirmed by an additional voxel-wise analysis of the mean phase shift values (Han et al. 2013). Overall, results on topographical differences of SWI abnormalities in MSA and PSP patients vary between studies, but large confirmative studies are warranted.

#### Neuromelanin imaging

Although NM-MRI to measure the volume and concentration of neuromelanin in the SN and LC is mainly used for the diagnosis of PD, only a few reports have used NM-MRI to study discrimination of atypical parkinsonism from PD (Kashihara et al. 2011; Matsuura et al. 2013; Ohtsuka et al. 2014). One study suggested that the volumes of the neuromelanin-positive region in the SNc of 28 patients with MSA, 11 patients with PSP, 10 patients with CBS and 54 patients with PD were reduced compared to those of 54 controls and 9 patients with SCA (Kashihara et al. 2011). When comparing 9 patients with MSA, 32 patients with PD and 23 controls with NM-MRI, signal intensities of the LC and SNc were decreased in MSA and PD patients, most prominently in the LC in MSA patients (Matsuura et al. 2013). Diagnostic accuracies, however, were not given in these two studies (Kashihara et al. 2011; Matsuura et al. 2013). A more recent study (Ohtsuka et al. 2014) studied NM-MRI in 53 patients who were clinically uncertain parkinsonism (including 30 patients who developed PD, 10 MSA-P and 13 PSP, respectively, after an observation period of at least 1.5 years) due to their early disease stage and 22 controls. Signal intensities of the lateral SNc were lower in the PD and MSA-P groups compared to the other groups and signal intensities of the LC were lower in the PD group compared to the other groups. Sensitivity and specificity of NM-MRI based on signal intensities of the lateral SNc and LC for discriminating PD from MSA-P were 60 and 90%, those for PD from PSP 63–88% and 77–92%, and those for MSA-P from PSP 80 and 85%, respectively. Results on signal changes on NM-MRI between patients with different degenerative parkinsonian disorders vary between studies, but large confirmative studies are warranted.

### Functional imaging techniques

#### rs-fMRI

Only few studies are available that explore functional connectivity with rs-fMRI in patients with atypical parkinsonism with none of them addressing diagnostic accuracy (Whitwell et al. 2011; You et al. 2011; Gardner et al. 2013).

Because neurodegenerative parkinsonian disorders cause different disease-specific widespread alterations of whole-brain circuitry, which may occur early on in the disease course (Holtbernd and Eidelberg 2014), rs-fMRI has the potential to identify highly specific networks separating the different neurodegenerative parkinsonian disorders.

#### ASL

There are no studies available exploring ASL-derived perfusion deficits in patients with APDs. However, because MSA and PSP patients show disease-specific perfusion deficits with FDG-PET (Holtbernd and Eidelberg 2014), ASL might have not only the potential to detect disease-specific perfusion in MSA and PSP, but also to identify atypical parkinsonism on an individual basis.

#### Magnetic resonance spectroscopy

Studies using 1H-MRS revealed reduced NAA/Cr and NAA/Cho ratios in the lentiform nucleus or striatum not only in atypical parkinsonism (Davie et al. 1995; Federico et al. 1997, 1999), but also in PD (Chaudhuri et al. 1996; Clarke and Lowry 2001; Firbank et al. 2002) as opposed to previously published results that suggested reduced striatopallidal NAA/Cr ratios only in MSA but not PD (Federico et al. 1997, 1999). Discrepancy between this study derives presumably due to technical factors including the application of different echo and relaxation times, voxel sizes and pulse sequences (Clarke and Lowry 2001; Firbank et al. 2002). By increasing sensitivity and dispersion of the chemical shift, use of higher magnetic field strengths in 1H-MRS may render this technique more important in the differential diagnosis of parkinsonian disorders, even though greater magnetic susceptibility may diminish this benefit (Esterhammer et al. 2010). Multiple regional single voxel 1H-MRS of the putamen, pontine basis and cerebral white matter at 3.0 T were applied in 24 patients with MSA compared to 11 PD patients and 18 healthy controls (Watanabe et al. 2004). While significant NAA/Cr ratio reductions in the pontine basis were observed in both cerebellar variant of MSA (MSA-C) and MSA-P, reduced putaminal NAA/Cr ratios were only found in MSA-P patients. There were significant NAA/Cr ratio reductions in the pontine basis as well as in the putamen in patients with MSA-P compared with both controls and PD, which suggests that the combined assessment of NAA/Cr ratios in the pontine basis and putamen may help distinguish MSA-P from PD; however, diagnostic accuracy values were not given in this study (Watanabe et al. 2004). While most of the 1H-MRS studies focussed the analysis on striatopallidal NAA/Cr ratios, more recently NAA/Cr ratios were determined from the left cerebellar hemisphere in 21 patients with PD, 21 with PSP, 15 with MSA (MSA-P *n* = 7 and MSA-C *n* = 8) and 14 controls. NAA/Cr ratios were significantly lower for the APDs compared to PD and controls allowing separation of PD from atypical parkinsonism with a sensitivity of 100%, a specificity of 64% and an overall diagnostic accuracy of 77% (Zanigni et al. 2015). This is in accordance with an earlier study, where both short- and long-echo-time (TE)MRS images showed significant decreases in NAA/Cr ratios in MSA-C and SCA2 compared to normal controls, though there was no difference between the two patient groups (Boesch et al. 2007).

Quantitative analysis techniques with 1H-MRSI have advantages over the alternative ratio-based methods, as the most commonly used standards, Cr and Cho, have been found to vary in concentration in some circumstances, making interpretation of ratios difficult in studies using 1H-MRS and being possibly one of the reasons for the conflicting results of 1H-MRS studies in neurodegenerative parkinsonism in the past (Clarke and Lowry 2001). Using MRSI at 1.5 T in 11 patients with PD, 11 with MSA-P, 6 with MSA-C, 13 with PSP and 18 controls, lower NAA concentrations in the pallidum, putamen and lentiform nucleus were revealed in patients with PSP and MSA-P compared to healthy controls and patients with PD (Guevara et al. 2010). A recent study also reported reduced NAA concentrations in 9 patients with MSA-P compared to healthy controls (Stamelou et al. 2015). Mainly due to the conflicting results, the limited specificity, and technical challenges of 1H-MRS, its use in the field of neurodegenerative parkinsonism is mainly experimental (Esterhammer et al. 2010).

### Multimodal imaging

Only few studies used multimodal imaging for distinguishing PD from atypical parkinsonism (Focke et al. 2011a; Barbagallo et al. 2016). The additive value of different MR techniques was studied to compare the nigrostriatal changes measuring volume, T2* relaxation rates, and mean diffusivity in nigrostriatal structures (SN, caudate nucleus, and putamen) of 26 patients with PD and 29 patients with MSA (including 16 patients with MSA-P) (Barbagallo et al. 2016). Patients with MSA had volume loss and both higher mean diffusivity values and T2* relaxation rates values in their putamina as well as also higher caudate mean diffusivity values compared to patients with PD, while there were no nigral changes between groups. A discriminant analysis showed that using T2* relaxation rates and mean diffusivity in the putamen, two measurements of microstructural damage allowed 96% accuracy to distinguish patients with MSA-P from those with PD (Barbagallo et al. 2016).

## Diagnosis of PD

PD is a slowly progressive neurodegenerative disease that begins years or even decades before onset of classical motor symptoms (Kalia and Lang 2015 ; Poewe et al. 2017). The clinical diagnosis of PD is a challenging exercise, with related disease entities such as ET, atypical parkinsonism or symptomatic parkinsonism often being confused, particularly in the early stages of the disease, when symptoms and signs are often insidious. Accuracy of a clinical diagnosis of the disease can be improved significantly by the stringent use of standard clinical criteria, such as the United Kingdom Parkinson’s Disease Society Brain Bank (UKPDSBB) criteria (Gibb and Lees 1988; Hughes et al. 2002; Tolosa et al. 2006; Poewe et al. 2017 ). Indeed, overall diagnostic accuracy of the UKPDSBB criteria has been estimated as 94% in a large neuropathological analysis (Hughes et al. 2002), while a recent meta-analysis including 20 studies with 11 of them using pathologic examination as gold standard revealed an overall diagnostic accuracy for the UKPDSBB of 83% (Rizzo et al. 2016a). This meta-analysis, however, did not account for the time aspect of the UKPDSBB criteria with hallmark features of atypical parkinsonism often occurring only in later disease stages. Indeed, the highest level of diagnostic accuracy can be reached through evaluation of a patient presenting with parkinsonism after symptom duration of 5 years (Adler et al. 2014; Rajput and Rajput 2014). The recently published new diagnostic criteria for PD by the International Parkinson and Movement Disorder Society (MDS) also include ancillary diagnostic tests as supportive criteria for a diagnosis of PD, which have a specificity greater than 80% for the differential diagnosis of PD from other parkinsonian conditions such as olfactory testing to demonstrate olfactory loss and MIBG-scintigraphy to document cardiac sympathetic denervation (Postuma et al. 2016). While structural imaging is suggested by the UKPDSBB criteria (Gibb and Lees 1988), MRI is not included in these criteria. However, MRI techniques now provide a range of opportunities to detect disease-related changes. There are several biomarkers to assess neurodegeneration, tissue microstructure, iron deposition, and brain function. Newer quantitative imaging techniques at high-field (3.0 T) and ultra-high-field (7.0 T) have recently been applied in patients with PD and have shown promising results in detecting abnormalities in the SN and nigrostriatal pathway using different MRI techniques in patients with PD. Table 5 summarizes the most relevant studies on higher field MRI to determine PD.

**Table 5:** Diagnostic accuracy of MR finding implemented on higher field MR systems (3.0 or 7.0 T) in PD

Reference	Cohort size	Main results	Discriminator	Accuracy, %
Iron-sensitive sequences				
Baudrexel et al. (2010)	PD 20/HC 20	Decreased T2* in SN bilateral Decreased T1 in SN controlateral	Decreased T1 in SN	Se 71 Sp 80 AUC = 0.75
Mahlknecht et al. (2017) (in press)	Meta-analysis including 364 patients with PD and 231 controls from 10 studies	Absence of DNH more common in PD than controls	Absence of DNH	Se 98 Sp 95
Multimodal imaging				
Menke et al. (2009)	PD 10/HC 10	Decreased SN volume Decreased VCDR Decreased SN volume + decreased VCDR	Combined SN volumetry with DTI of SN	Se 100 Sp 80
Peran et al. (2010)	PD 30/HC 22	Increased R2* in the SN Reduced FA in the SN Increased mean diffusivity in the putamen or caudate nucleus	Multimodal MRI study at 3.0 T using a combination of different MR markers including volumetry, mean R2*, mean diffusivity and FA applied in 6 deep grey matter structures (SN, red nucleus, thalamus, putamen, caudate, pallidum)	Combinations of three markers achieved a maximum AUC = 0.98 Combinations of four markers achieved a maximum AUC = 0.99
Du et al. (2011)	PD 16/HC 16	Better discrimination of combined use of transverse relaxation rate and FA values in the SN compared to transverse relaxation rate or FA alone	Multimodal imaging using a combination of transverse relaxation rate and FA values in the SN	AUC = 0.99
			Transverse relaxation rate in the SN	AUC = 0.93
			FA in the SN	AUC = 0.74
Long et al. (2012)	PD 19/HC 27	The combination of multimodal imaging and multi-level measurements provided good diagnostic accuracy ↓ Regional homogeneity value in the bilateral middle frontal gyrus, orbital part, ↓ amplitude of low-frequency fluctuations decreases in the left rolandic operculum in PD vs. HC ↑ Regional functional connectivity strength in the left parahippocampal gyrus, left angular gyrus and right middle temporal gyrus in PD vs. HC ↓ GM volume in the left paracentral lobule in PD vs. HC ↑ GM volume of the left precentral gyrus and the bilateral posterior cingulate gyrus in PD vs. HC Brain regions showing WM volume changes were mainly located in the frontal and temporal lobes	Automated method combining resting state fMRI and structural images	Se 79 Sp 93
Diffusion imaging				
Prodoehl et al. (2013)	PD 15/MSA 14-P/PSP 12/ ET 14/HC 17	Multi-target imaging approach focused on the basal ganglia and cerebellum accurately classifies control subjects and patients with PD, MSA-P, PSP, and ET	Model using DTI measures from the caudate nucleus and SN, and dentate nucleus	Se 92 (PD) Sp 87 (ET) AUC = 0.96
			Model using DTI measures from the SN and MCP	Se 94 (PD) Sp 100 (PD vs. MSA) AUC = 0.99
			Model using DTI measures from the putamen and SN	Se 87 (PD) Sp 100 (PD vs. PSP) AUC = 0.96
			Model using DTI measures from the putamen, SN, and dentate nucleus	Se 90 (PD) Sp 100 (PD vs. MSA, PSP) AUC = 0.99
Hirata et al. (2016)	Meta-analysis including 806 PD patients and 626 controls from 22 studies	↓ Nigral FA in PD	Decreased nigral FA	Se 72 Sp 63
Neuromelanin-sensitive MRI				
Castellanos et al. (2015)	PD 36 (23 idiopathic and 13 monogenic PARKIN or LRRK2 mutations)/HC 37	↓ Signal intensity in the locus coeruleus and SNc in neuromelanin-sensitive imaging in PD vs. HC	SNc volume measurements	Se 92 (PD) Sp 89 (vs. HC) AUC = 0.92
Resting state fMRI				
Szewczyk-Krolikowski et al. (2014)	PD 19/HC 19 in the discovery cohort PD 13 (including 5 drug naïve) in the validation cohort	Reduced functional connectivity within BGN in PD	Average BGN connectivity	Se 100 (PD-discovery cohort), 85 (PD-validation cohort) Sp 89
Chen et al. (2015)	PD 21/HC 26	The majority of the most discriminative functional connections were located within or across the default mode, cingulo-opercular and frontal-parietal networks and the cerebellum.	Whole-brain functional connectivity	Accuracy 94
Wu et al. (2015)	PD 58/HC 54 (PD 28/HC 28 in the derivation sample and PD 30/HC 26 in the validation sample)	The topographic pattern of neural activity in PD was characterized by decreased activity in the striatum, supplementary motor area, middle frontal gyrus, and occipital cortex, and increased activity in the thalamus, cerebellum, precuneus, superior parietal lobule, and temporal cortex	PD-related spatial covariance pattern-amplitude of low-frequency fluctuation	Whole cohort: Se 91 (PD) Sp 89 (HC) AUC = 0.97 Derivation sample: Se 82 Sp 79 AUC = 0.92 Validation sample: Se70 Sp 69 AUC = 0.78

*T* tesla, *MRI* magnetic resonance imaging, *Se* sensitivity, *Sp* specificity, *AUC* area under the curve, ↑ significant higher, ↓ significant lower, *PD* Parkinson’s disease, *HC* healthy controls, *ET* essential tremor, *MSA-P* parkinsonian variant of MSA, *PSP* progressive supranuclear palsy, R2* relaxation rates = 1/T2*, *BGN* basal ganglia network, *SN* substantia nigra, *SNc* SN pars compacta, *DNH* dorsolateral nigral hyperintensity, *GM* grey matter, *WM* white matter, *DWI* diffusion-weighted imaging, *DTI* diffusion tensor imaging, *FA* fractional anisotropy, *VCDR* voxels for all connectivity-defined subregions, *MCP* middle cerebellar peduncle, *fMRI* functional MRI

### Structural MRI with conventional MRI sequences

While structural brain imaging using MRI at 1.5 T is usually normal in patients with uncomplicated early PD, in more advanced stages of the disease, signal changes in the area of the SN such as hyperintensities in T2-weighted sequences or smudging of the red nucleus borders towards the SN may occur (Hotter et al. 2009; Mahlknecht et al. 2010). Interestingly, a study at 1.5 T exploring signal intensities of the basal ganglia including the SN in 70 patients with PD, 170 controls and 38 patients with atypical parkinsonism (MSA, *n* = 11; PSP, *n* = 22; CBS, *n* = 5) found that signal alterations of SN and globus pallidus internus in structural MRI with conventional MRI sequences separated all parkinsonian patients from controls with a sensitivity of 86% and a specificity of 90% (Jesse et al. 2012). Recently, a new MRI finding distinguishing between PD patients and healthy controls has been described in the SN using iron-sensitive MRI sequences at higher field strengths at 3.0 and 7.0 T. Controls consistently display a hyperintense, ovoid area within the dorsolateral border of the otherwise hypointense SNc, which seems to correspond to nigrosome-1 based on a post-mortem 7.0 T MRI study with histopathological correlation (Blazejewska et al. 2013). Because nigrosome-1 is a histological concept, which refers to a calbindin-negative subregion in the SNc, the descriptive terms ‘Dorsolateral Nigral Hyperintensity’ (DNH) or ‘Nigral hyperintensity’ have been introduced (Reiter et al. 2015; Bae et al. 2016). Overall, in the studies published so far, loss of DNH had a high sensitivity (79–100%) and specificity (85–100%) to separate PD from controls (Schwarz et al. 2014; Reiter et al. 2015; Bae et al. 2016; Kim et al. 2016; Sung et al. 2016). Moreover, a recent meta-analysis including 364 patients with PD and 331 controls from 10 studies showed an overall sensitivity and specificity of the absence of DNH for PD vs. controls of 98 and 95% and of 95 and 94% when including studies performed at 3.0 T only (Mahlknecht et al. 2017). This meta-analysis demonstrates a potential value in differentiating PD from uncertain movement disorders such as DIP, ET and dystonic tremor. Indeed, a recent study reported that patients with DIP could be discriminated from those with PD with high sensitivity and specificity (Sung et al. 2016), while loss of DNH seems not to discriminate between PD and atypical parkinsonism due to MSA and PSP (Reiter et al. 2015; Bae et al. 2016; Kim et al. 2016).

As NM-MRI can be used to detect SN changes in early stage PD by visual inspection, it may become a useful tool in clinical practice (Reimao et al. 2015a, b).

### Quantitative MRI

#### Quantitative assessment of atrophy

While patients with atypical parkinsonism may show regional atrophy in the basal ganglia and infratentorial structures by different ROI-based approaches as discussed above, regional volumes in these regions are usually normal in early stage PD (Mahlknecht et al. 2010; Holtbernd and Eidelberg 2014).

Two recent meta-analyses (Shao et al. 2015; Yu et al. 2015) investigated volume changes in patients with PD compared to controls using a plethora of studies (Burton et al. 2004; Cordato et al. 2005; Nagano-Saito et al. 2005; Summerfield et al. 2005; Beyer et al. 2007; Ramirez-Ruiz et al. 2007; Karagulle Kendi et al. 2008; Camicioli et al. 2009; Jubault et al. 2009; Martin et al. 2009; Pereira et al. 2009; Sanchez-Castaneda et al. 2009; Tir et al. 2009; Dalaker et al. 2010; Kostic et al. 2010; Nishio et al. 2010; Cerasa et al. 2011; Focke et al. 2011a, b; Meppelink et al. 2011; Compta et al. 2012; Fernandez-Seara et al. 2012; Hong et al. 2012; Ibarretxe-Bilbao et al. 2012; Tessitore et al. 2012a ; Lee et al. 2013a; O’Callaghan et al. 2013; Ellfolk et al. 2014; Menke et al. 2014; Sehm et al. 2014) using VBM. Reduced GM volume was noted in the frontal lobe including bilateral middle and inferior frontal gyri and the left precentral gyrus, the parietal lobe including the left superior parietal lobule and precuneus, the occipital lobe including bilateral cuneus, and the limbic lobe including the left anterior cingulate in one meta-analysis (Shao et al. 2015). Intriguingly, when analysing only studies on patients with disease duration within 3 years, only the left limbic lobe, left parietal lobe and bilateral occipital lobe atrophy were found (Shao et al. 2015). The other meta-analysis demonstrated significant convergence in a predominantly anterior cortical distribution, with the largest cluster situated in the right inferior frontal gyrus (Yu et al. 2015). However, studies using this atrophy patterns as diagnostic marker for early PD are lacking.

There are, however, several attempts to assess volumes of the SN in PD. Volumes of SNc have been measured using multispectral structural MRI at 3.0 T creating a weighted mean of multiple echoes (from multiecho T1-weighted, multiecho proton density, T2-weighted, and T2-weighted FLAIR sequences) in 29 patients with PD and 27 control subjects. Indeed patients with PD had significantly decreased SNc volumes; however, diagnostic accuracy was not given in this study (Ziegler et al. 2013). An earlier study, investigating a novel high-resolution volumetric method based on a single-pulse observation of T1 revealed significantly smaller whole SN volumes in PD patients compared with healthy subjects at 3.0 T (Menke et al. 2009). Whereas diagnostic accuracy to differentiate PD vs. healthy controls was suboptimal (sensitivity 80%, specificity 70%) for SN volumes, combining SN volumetry and its connectivity with the thalamus via DTI (see the section “Multimodal imaging”) improved the classification sensitivity to 100% and specificity to 80% for PD (Menke et al. 2009). Moreover, NM-MRI can be used to measure SN volumes (see the section “Neuromelanin imaging”) (Castellanos et al. 2015; Langley et al. 2016).

MRI studies at 1.5 T have shown that morphological changes including volume loss in the basal ganglia or signal changes in the SN can be detected in advanced PD (Hotter et al. 2009; Mahlknecht et al. 2010). This raises the possibility that greater sensitivity of MRI at higher magnet fields complemented by higher tissue contrast may lead to more robust findings of structural abnormalities in early PD. Due to the increased SNR and impressive anatomic delineation that is provided by high-field scanning, sensitivity of atrophy measures may increase. MRI at higher field strengths leads to a better grey-to-white-matter contrast, showing sharp images and smooth transitions between the different brain structures. Indeed, an interesting approach to investigate changes in local volumes is subcortical nuclei shape analysis based on T1 imaging at 3.0 T (Sterling et al. 2013; Menke et al. 2014; Nemmi et al. 2015). When comparing 21 PD patients and 20 control subjects using GM density and subcortical nuclei volume and shape, volume differences in the putamen and shape differences in the putamen and the caudate nucleus between the two groups have been found. Using discriminant analysis using variable combinations of these changes, PD patients could be discriminated from controls with an accuracy ranging between 75 and 83% (Nemmi et al. 2015). Moreover, a pilot study using ultra-high-field MRI with 7.0 T has demonstrated that the increased SNR may help to better delineate the SN and assess shapes and boundaries of the SN when using T2*-weighted gradient echo sequences. While controls had a smooth ‘‘arch’’ shape lateral boundary of the SN, the lateral boundary of the SN was serrated in PD patients. By quantifying these differences of the lateral boundaries of the SN via an undulation value, PD patients had significant higher values compared to the controls with only a small overlap of the individual values between the groups (Cho et al. 2011).

### Quantitative structural MR-based techniques

#### Diffusion imaging

Multiple studies have been performed to study the usefulness of DTI measures in the SN for the diagnosis of PD, and several meta-analyses have been performed exploring its diagnostic potential (Cochrane and Ebmeier 2013; Schwarz et al. 2013; Hirata et al. 2016). A notable effect size of −0.64 was found for lowered FA in the SN for patients with PD vs. controls in one meta-analysis on 9 studies with a total of 193 patients with PD and 195 controls (Cochrane and Ebmeier 2013), although its discriminatory capability to diagnose PD in a further more recent meta-analysis on 22 studies including 806 PD patients and 626 controls was insufficient with a pooled sensitivity and specificity of 72 and 63%, respectively (Hirata et al. 2016). When combining nigral FA, however, with other quantitative MR parameters sensitive to complementary tissue characteristics (i.e. multimodal MRI), better discrimination compared with the single markers alone could be achieved (Peran et al. 2010; Du et al. 2011; Focke et al. 2011a).

A further study applying a multi-target DTI approach using different DTI metrics (fractional anisotropy, radial diffusivity, longitudinal diffusivity, and mean diffusivity) focused on the basal ganglia and cerebellum in 15 patients with PD and 12 patients with ET found that DTI measures from the caudate and substantia nigra separated PD from essential tremor (ET) with a sensitivity of 92% and a specificity of 87% (Prodoehl et al. 2013).

Using diffusion imaging at 1.5 T, abnormalities within the olfactory bulb have been reported consistently, which have been associated with decreases in olfactory performance (Scherfler et al. 2006, 2013; Rolheiser et al. 2011; Agosta et al. 2013). Moreover, by applying voxel-wise analysis on diffusivity maps, changes of the olfactory tract derived from a derivation sample of each 12 patients with PD and controls allowed to correctly discriminate 17 independent individuals of a test cohort (9 patients with PD and 8 controls) with a sensitivity of 100% and a specificity of 88% (Scherfler et al. 2006).

Advanced diffusion post-processing techniques using bi-tensor modelling of diffusion imaging sequences such as FW imaging (Ofori et al. 2015a, b) and NODDI (Kamagata et al. 2016) seem to produce more reliable results to differentiate PD from non-PD. Indeed, a study from the Parkinson’s Progressive Marker Initiative (PPMI) did not find differences in FA values within the SN in PD patients compared with controls (Schuff et al. 2015), while another study using a subgroup of the subjects from the PPMI cohort exploiting a bi-tensor model found increased FW measurements within the SN compared with healthy controls (Ofori et al. 2015a). With the use of NODDI, evaluation of nigrostriatal changes through detecting the microstructure of dendrites is possible and more specific than standard DTI indices (Zhang et al. 2012). A recent study showed that the mean FAorientation dispersion index (OD), and intracellular volume fraction (Vic) in the contralateral SNc and mean OD in the contralateral putamen in PD patients are significantly lower than those in healthy controls, and the Vic of the contralateral SNc was the best parameter for discriminating PD from controls, with a sensitivity of 0.88 and a specificity of 0.83 (Kamagata et al. 2016).

Tractography can be used to calculate diffusion measures (MD, FA) and connectivity measures can be calculated, and with generated DTI data, it is possible to assess whether there are changes in anatomical connectivity in PD patients (Menke et al. 2009; Sharman et al. 2013; Ziegler et al. 2014; Zhang et al. 2015). Reduced connectivity between the SN with the striatum and thalamus has been reported for PD patients compared with control (Menke et al. 2009; Ziegler et al. 2014; Zhang et al. 2015), but studies exploring the diagnostic accuracy of structural connectivity changes are warranted.

An interesting approach in analysing diffusion images to detect patients with PD at the individual level has been explored by performing a pattern-recognition analysis (Haller et al. 2012). Group-level TBSS of 3.0 T diffusion images and individual level SVM classification were analysed for 40 patients presenting with clinically uncertain parkinsonism, of whom probable diagnoses could be made in 35 patients after at least 2.5 years (PD in 17, MSA in 5, vascular parkinsonism in 3, DLB, DIP and atypical tremor in 2 each, and other diagnoses including PSP in 1 each). At the group level, patients with PD vs. non-PD parkinsonism or tremor disorder had spatially consistent increase in FA and decrease in RD and MD in a bilateral network, predominantly in the right frontal white matter, while at the individual level, SVM correctly classified patients with PD at the individual level with accuracies up to 97% using tenfold cross-validation. No validation and test cohorts were used in this study, but confirmative studies are warranted.

#### Magnetization transfer imaging

Promising preliminary results concerning detection of the nigral alteration in PD were recently obtained by application of magnetization transfer imaging that demonstrated decreased MTR in the substantia nigra of PD patients (Tambasco et al. 2003; Eckert et al. 2004; Anik et al. 2007; Mahlknecht et al. 2010), but confirmatory studies exploring diagnostic accuracy of abnormal nigral MTRs are warranted.

#### Iron-sensitive techniques

Using quantitative iron-sensitive techniques, nigral changes due to increased iron content such as R2*, phase imaging and QSM have been consistently reported in PD using high-field MRI as well as 1.5 T (Martin et al. 2008; Baudrexel et al. 2010; Du et al. 2012; Esterhammer et al. 2015; Pyatigorskaya et al. 2015; Azuma et al. 2016; Hopes et al. 2016; Tuite 2016). Consistently, there has also been reported overlap with healthy controls. Interestingly, it seems that PD patients with an advanced disease duration might have lower nigral relaxation rate R2* values than PD patients with an earlier disease duration, which has been explained by the hypothesis that neuronal degeneration with consecutive gliosis might lengthen T2 relaxation times within the tissue and thus counteract the increase of relaxation rates in PD (Esterhammer et al. 2015). This could also explain that some authors have found no change between nigral alterations reflecting increased iron content using quantitative iron-sensitive techniques between PD and controls (Aquino et al. 2014; Dashtipour et al. 2015; Reimao et al. 2016), while most of the studies did (Martin et al. 2008; Du et al. 2012; Esterhammer et al. 2015; Pyatigorskaya et al. 2015; Azuma et al. 2016; Hopes et al. 2016). Furthermore, a comparative study of R2* and QSM showed that QSM had higher sensitivity for displaying PD-related changes in the SNc and correlated better with clinical parameters than R2*, suggesting the high potential of QSM as a biomarker of iron-related pathology in PD (Du et al. 2016).

When combining nigral iron measures, however, with other quantitative MR parameters sensitive to complementary tissue characteristics (i.e. multimodal neuroimaging/MRI), better discrimination compared with the single markers alone could be achieved (Peran et al. 2010; Du et al. 2011).

A further quantitative approach using R2* at ultra-high-field MRI has been used to quantify the SN shape (Cho et al. 2011; Kwon et al. 2012). A high diagnostic accuracy on distinguishing PD from controls can be achieved using a method quantifying the lateral boundaries of the SN, which resembles a smooth “arch” shape on the lateral boundary of the SN in healthy controls and which was more serrated in PD (Cho et al. 2011).

#### Neuromelanin-sensitive MRI and other T1-based techniques

NM-MRI sequences can reveal signal changes in the SN. Reduced size and signal intensity of the SN were reported in PD patients using NM-MRI with a high diagnostic accuracy (Castellanos et al. 2015). Interestingly, also the LC showed reduced signal intensity in NM-MRI in PD patients compared with controls (Sasaki et al. 2006). When comparing SNc and LC volumes using an automated neuromelanin aiming diagnostic tool, diagnostic accuracy was better when using SNc volume than LC volume to separate 36 PD patients from 37 healthy controls. Contralateral atrophy in the SNc showed the highest power to discriminate PD patients from controls with an AUC of 0.93–0.94 providing a sensitivity of 91–92% and a specificity of 89% (Castellanos et al. 2015).

More recently, diagnostic accuracy of NM-MRI was studied in 15 ET patients, 12 drug-naïve PD patients as well as 10 age-matched control subjects (Nakamura and Sugaya 2014). In the PD group, the area and width of the T1 high signal in the SN region were significantly decreased compared with the ET and age-matched controls discriminating early stage PD from ET with a sensitivity of 66.7% and a specificity of 93.3% (Nakamura and Sugaya 2014). Another study assessed 39 PD patients and 30 control subjects in a prospective case–control study to investigate the pattern of neuromelanin signal intensity loss within the SNc, LC, and ventral tegmental area (Seidel et al. 2015). A prominent reduction of normalized neuromelanin volume in the posterior SNc was found in the PD group, which allowed the best differentiation of patients with PD and control subjects, followed by the anterior SNc and the LC. Measures of diagnostic accuracy, however, were not reported in this study.

Recently, a multimodal imaging study used a novel approach to analyse iron deposition via SWI in ROIs of the SNc defined by NM-MRI (see section “Multimodal imaging”) (Langley et al. 2016). Because visual inspection of NM-MRI images by experienced neuroradiologists provides results comparable to quantitative analyses in the detection of SN changes in early stage PD, NM-MRI may become a useful tool in clinical practice (Reimao et al. 2015a). Because pathological changes in the SN occur also in atypical parkinsonism, NM-MRI might not represent a tool to discriminate among different forms of degenerative parkinsonian syndromes, although a recent study suggests that quantitative analysis of NM-MRI of SN regions could be possibly helpful for the differential diagnosis of degenerative parkinsonian disorders (Ohtsuka et al. 2014). Confirmative studies are, however, warranted.

Also other T1-based quantitative MRI techniques have been used in PD patients. Using combined T1- and T2*-mapping, voxel-wise changes of local relaxation times have been studied in the midbrain and lower brainstem of early PD compared to controls (Baudrexel et al. 2010). Interestingly, in accordance with the NM-MRI studies, a widespread reduction of midbrain T1 values contralateral to the clinically more severely affected side has been found, exceeding the SN and reaching non-dopaminergic areas in the pontomesencephalic junction potentially involved in early non-motor symptoms of PD. The reduced T1 values in the caudal SN separated the PD patients from controls with an AUC of 0.75.

When using the inversion recovery ratio at 1.5 T, another T1-based quantitative MRI technique, signal loss in the SN has also been reported in patients with PD (Hutchinson and Raff 1999, 2008; Hu et al. 2001; Hutchinson et al. 2003; Minati et al. 2007). Some studies were able to completely discriminate between PD patients and healthy controls (Hutchinson and Raff 1999; Hutchinson et al. 2003), while other authors found an overlap between normal and PD values in their results (Hu et al. 2001; Minati et al. 2007), but further confirmatory studies on higher field MRI are warranted.

### Functional imaging techniques

#### rs-fMRI

rs-fMRI has been applied as a non-invasive tool in numerous studies to assess functional abnormalities observed in PD in the resting state (that is without the effects of particular motor or cognitive tasks) (Tessitore et al. 2012 b, c; Agosta et al. 2014; Canu et al. 2015; Tahmasian et al. 2015). In PD, decreased coupling was reported in the cortico-striatal sensorimotor network and between the striatum and the brainstem structures as well as increased coupling in the associative network, most probably compensatory (Tahmasian et al. 2015). Indeed, a very recent meta-analysis including 10 studies reporting 11 comparisons in 212 patients with PD and 182 controls demonstrates a consistent and coexistent pattern of impairment and compensation of intrinsic brain activity that predominantly involves the default mode and motor networks (Pan et al. 2017).

Moreover, rs-fMRI has been also applied to identify specific networks separating PD from controls (Szewczyk-Krolikowski et al. 2014; Chen et al. 2015; Wu et al. 2015). Functional connectivity within the basal ganglia network (BGN) derived from 80 elderly HC participants was used in a group of 19 patients with early PD compared to 19 HCs to identify a threshold for group separation, which was then applied in a validation cohort of 13 PD patients. Diagnostic accuracy was 95% to separate PD from controls in the derivation cohort and 85% in the validation cohort, respectively (Szewczyk-Krolikowski et al. 2014). By applying whole-brain resting-state functional connectivity patterns (derived from functional connectivity between each pair of 116 ROIs derived from a prior atlas) followed by SVM classification, 21 patients with PD were compared to 26 controls. The majority of the most discriminative functional connections were located within or across the default mode, cingulo-opercular and frontal-parietal networks and the cerebellum. This SVM classifier using these disease-related resting-state network alterations achieved a classification accuracy of 93.6% using leave-one-out cross-validation (Chen et al. 2015). Another approach aimed to identify a disease-related spatial covariance pattern of spontaneous neural activity in a derivation sample of 28 PD patients and 28 controls and a validation cohort of 30 PD patients and 26 controls. The topographic pattern of neural activity in PD was characterized by decreased activity in the striatum, supplementary motor area, middle frontal gyrus, and occipital cortex, and increased activity in the thalamus, cerebellum, precuneus, superior parietal lobule, and temporal cortex. This pattern expression was elevated in the patients with PD compared to the controls, with diagnostic accuracies of 80, 73 and 90% in the derivation, the validation and the whole cohort, respectively (Wu et al. 2015).

Overall, high diagnostic accuracies in separating PD patients from healthy controls have been reported for these rs-fMRI studies using different approaches of analyses (Szewczyk-Krolikowski et al. 2014; Chen et al. 2015; Wu et al. 2015), but confirmatory studies are warranted.

#### Arterial spin labelling

Several studies on ASL-MRI have consistently shown symmetrical cortical hypoperfusion in PD involving predominantly the parieto-occipital areas and the dorsolateral prefrontal cortex (Wolf and Detre 2007; Kamagata et al. 2011; Melzer et al. 2011; Fernandez-Seara et al. 2012; Madhyastha et al. 2015). In PD patients with dementia, posterior perfusion deficits were found to be more striking than in PD without dementia cases (Kamagata et al. 2011). Another study using both FDG-PET metabolism and ASL-MRI perfusion found overlapping metabolic and perfusion deficits in PD (Teune et al. 2014). Therefore, because PD patients show disease-specific metabolism patterns with FDG-PET characterized by relatively increased metabolism in the globus pallidus and putamen, thalamus, cerebellum, pons, and sensorimotor cortex and relative decreases in the lateral frontal and parieto-occipital areas (Eckert et al. 2007; Meles et al. 2017), ASL-MRI has the potential to identify PD early in the disease course.

#### MRS

In PD, 1H-MRS studies have reported reduced NAA as well as elevated lactate and choline, while other studies failed to detect these changes (Bowen et al. 1995; O’Neill et al. 2002; Esterhammer et al. 2010; Guevara et al. 2010; Brockmann et al. 2012; Emir et al. 2012; Levin et al. 2014; Weiduschat et al. 2015). More recently, three-dimensional high-field MRSI of the SN region was applied in 20 patients with established PD, 10 with atypical parkinsonism and 22 controls. Differences in rostral to caudal nigral NAA/Cr ratios were significantly different between PD patients and both controls and patients with atypical parkinsonism. The reversed rostral to caudal NAA/Cr ratios in PD patients allowed a discrimination from both controls and patients with atypical parkinsonism with a high diagnostic accuracy (Groger et al. 2013), but confirmative studies in earlier disease stages are warranted.

### Multimodal imaging

There are various multimodal imaging studies in PDon combinations of volumetry, R2*, MD, or FA (Menke et al. 2009; Peran et al. 2010; Du et al. 2011), showing that combinations of different methods and techniques sensitive to complementary tissue characteristics may provide better differentiation than single methods and techniques. As a multimodal technique, various MRI sequences can be combined to enhance the diagnostic work-up for PD (Menke et al. 2009, 2010; Peran et al. 2010; Du et al. 2011; Long et al. 2012).

Using a multimodal approach, one study showed a 95% accuracy in the discrimination of PD compared with healthy controls using several combinations of R2* and FA in the SN, and MD in the striatum (Peran et al. 2010). Using a combination of multimodal imaging and multi-level measurements, 19 early PD could be distinguished from 27 healthy controls with an accuracy of 87% (Long et al. 2012). A further study combined SN volumetry (see the section “Quantitative assessment of atrophy”) with DTI estimated connectivity profile resulting from running probabilistic tractography at 3.0 T (Menke et al. 2009, 2010). Whereas SN volume could discriminate between PD patients and controls with considerable overlap of volumes between groups (sensitivity 80%, specificity 70%), mean FA for the whole SN failed to discriminate patients from healthy controls even on a group level. Nevertheless, by combining SN volumetry and its connectivity with the thalamus via DTI, classification sensitivity was improved to 100% and specificity to 80% for PD, respectively (Menke et al. 2009). The combination of transverse relaxation rate and FA measures in the SN of PD showed high precision in distinguishing PD and healthy controls (Du et al. 2011). A more recent multi-contrast study including 28 patients with PD and 54 controls analysed iron deposition via SWI in regions of the SNc defined by NM-MRI (Langley et al. 2016). Using such an approach, significantly more hypointense signal in the SWI sequences was observed in the SNc defined by NM-MRI in the PD group compared to the controls with the lateral ventral region of the SNc exhibiting the greatest increase of hypointensity and having the greatest potential to discriminate PD from controls. No diagnostic accuracy measures, however, were reported in this study.

## Conclusion and future development

**Fig. 3:**
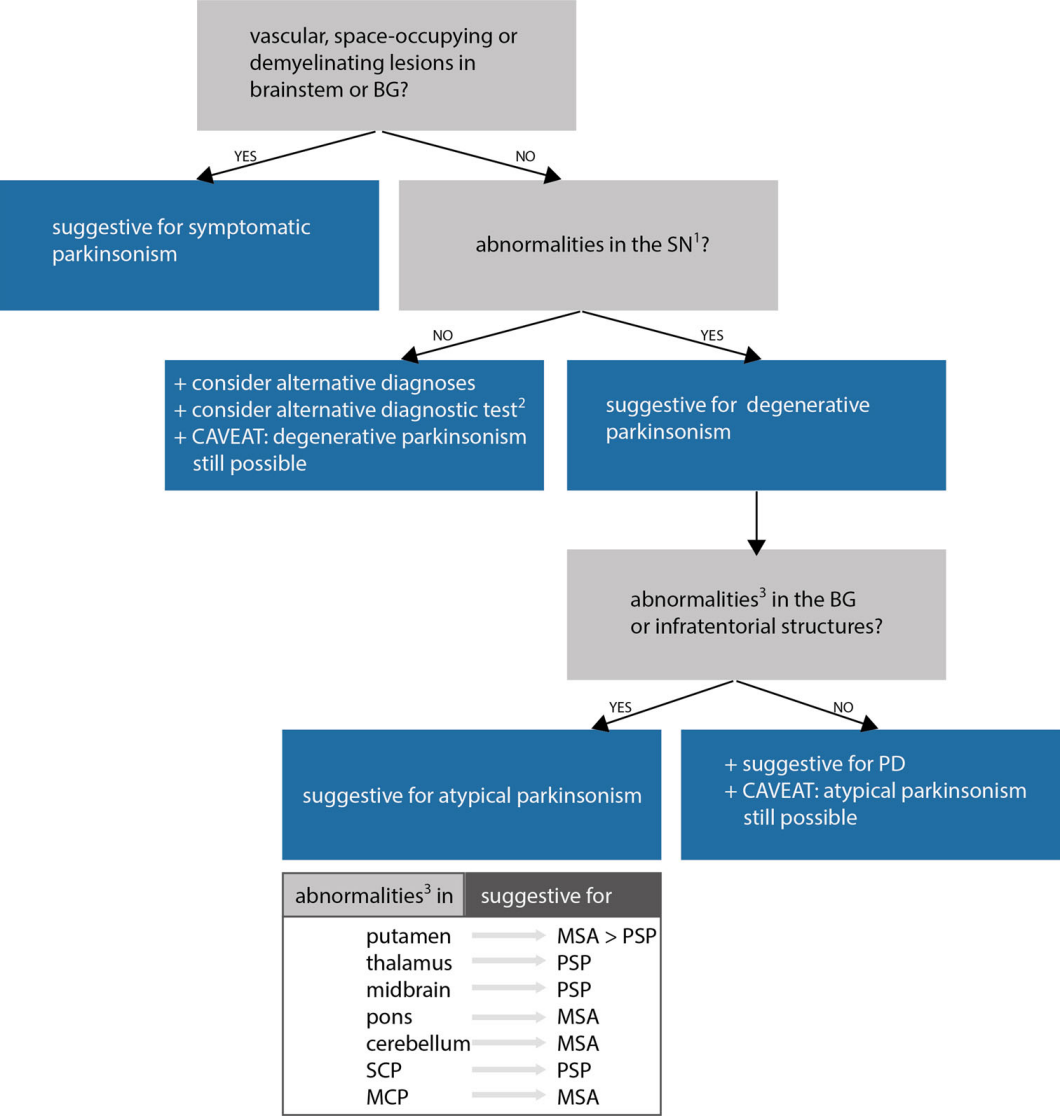
Pragmatic approach to reading a MRI in a patient presenting with early parkinsonism. *1* With higher field MRI using neuromelanin-sensitive MRI or iron-sensitive sequences (lack of DNH). *2* Such as radiotracer-imaging studies (e.g. presynaptic dopaminergic imaging such as dopamine-transporter-SPECT or myocardial postganglionic sympathetic imaging such as metaiodobenzylguanidine-scintigraphy). *3* Refer to qualitative (such as atrophy or signal changes) or quantitative changes (using quantitative assessment of regional cerebral atrophy or quantitative structural MR-based techniques such as diffusion imaging or iron-sensitive sequences). *BG* basal ganglia, *SN* substantia nigra, *PD* Parkinson’s disease, *MSA* multiple system atrophy, *PSP* progressive supranuclear palsy, *SCP* superior cerebellar peduncle, *MCP* middle cerebellar peduncle

Since 1986 when two MRI studies on neurodegenerative “Parkinson plus syndromes” were published (Pastakia et al. 1986; Drayer et al. 1986), MRI has become a well-established method that can be used for the diagnostic work-up of parkinsonism in clinical routine, providing specific information that points toward the diagnosis of a neurodegenerative condition. The role of MRI has progressed from excluding symptomatic parkinsonism due to other pathologies to distinguishing PD from APD based on specific changes in the basal ganglia and infratentorial structures (Mahlknecht et al. 2010). Figure 3 gives a pragmatic approach for reading an MRI in a patient presenting with early parkinsonism, while Table 6 summarizes useful MRI findings to help clinicians diagnose patients presenting with degenerative parkinsonism. Only over the past decade, advances in MR methodology allowed the detection of PD-related MR changes and provided a boost for the diagnosis of early PD. Advanced imaging techniques at 3.0 T or higher field strengths have recently been applied in patients with PD and have shown promising results in detecting abnormalities in the SN, nigrostriatal pathway and outside the nigrostriatal system as summarized in this review using diffusion imaging, NM-MRI, iron-sensitive sequences, 1H-MRSI, rs-fMRI and multimodal imaging in patients with PD. The most consistently reported abnormalities in PD include loss of DNH (Schwarz et al. 2014; Reiter et al. 2015; Bae et al. 2016) and nigral neuromelanin signal changes (Kashihara et al. 2011; Matsuura et al. 2013; Ohtsuka et al. 2014; Castellanos et al. 2015; Reimao et al. 2015a, b; Langley et al. 2016) establishing these qualitative MR markers in routine clinical practice for the diagnosis of early PD (Lehericy et al. 2017). There are also promising quantitative markers including QSM, multiecho susceptibility map-weighted imaging, adiabatic techniques T1rho, T2rho, relaxations along a fictitious field (RAFF), NM-MRI, as well as post-processing diffusion imaging techniques including FW or NODDI (Barbosa et al. 2015; Ofori et al. 2015a, b; Du et al. 2016; Kamagata et al. 2016; Langkammer et al. 2016; Nam et al. 2016; Planetta et al. 2016). Limitations of these techniques, however, include their unavailability on most conventional scanners and the lack of normative databases (Lehericy et al. 2017). Combination of different markers sensitive to complementary tissue characteristics may evolve to assist in the differential diagnosis of degenerative Parkinsonism in clinical practice including volume measurements as well as diffusion and iron measurements in infratentorial structures, SN and basal ganglia (Peran et al. 2010; Du et al. 2011; Esterhammer et al. 2015; Barbagallo et al. 2016; Tuite 2016).

**Table 6:** Summary on characteristic MRI patterns for the differential diagnosis of neurodegenerative parkinsonism

	PD	MSA(-P)	PSP
cMRI			
Normal	++	–/+	–/+
Putaminal atrophy	–	++/+++	++
Putaminal hyperintense rim^a^	+	++	+
Putaminal hypointensity on T2^a^	–	++	–
Atrophy of pons and vermis cerebellaris	–	++	+
Signal changes in the pons (hot cross bun sign) or MCPs^a^	–	++	–
Midbrain atrophy Mickey mouse sign and king-penguin silhouette	–	–	++
MRI planimetry			
Midbrain diameter	–	+	++/+++^b^
Decreased m_d_/p_d_ ratio	–	+	+++
Decreased m_a_/p_a_ ratio	–	+	+++
Increased MRPI	+	–	+++
Diffusion imaging			
Increased putaminal diffusivity	–	+++	++
Increased diffusivity of MCP	+	+++	+
Increased diffusivity of SCP	–	–	+++
Iron-sensitive sequences			
Increased putaminal iron load (e.g. putaminal hypointensity on T2* and SWI)^c^	+	+++^d^	++

–, <20%; +, 20–50%; ++, 50–70%; +++, 70–90%; ++++, >90%. Signal changes (hyper- and hypointensities) refer to T2-weighted sequences. m_d_/p_d_ ratio = ratio of midsagittal midbrain to pons diameter by placing midsagittal elliptical ROIs. m_a_/p_a_ ratio = ratio of midsagittal midbrain area to pons area. MRPI = MR-Parkinsonism index = (area pons/area midbrain) × (width MCP/width SCP)

*cMRI* conventional magnetic resonance imaging with routine sequences, *T* tesla, *PD* Parkinson’s disease, *MSA* multiple system atrophy, *MSA-P* parkinsonian variant of MSA, *PSP* progressive supranuclear palsy, *MCP* middle cerebellar peduncle, *MRPI* MR Parkinsonism Index, *SCP* superior cerebellar peduncle, *ROI* region of interest, *SWI* susceptibility-weighted imaging

^a^At 1.5 T

^b^Depending on the assessment (for further details see text)

^c^Depending on the sequence used to measure iron load (for further details see text)

^d^Typically iron deposition in lower outer part of putamen

As summarized in this review, changes in the putamen on diffusion imaging or iron-sensitive imaging are typically present in MSA and not in early stage PD. Moreover, the development of classifiers applied to different MR methodologies may also help clinicians to differentiate between these conditions (Haller et al. 2012; Castellanos et al. 2015; Chen et al. 2015; Huppertz et al. 2016; Planetta et al. 2016; Scherfler et al. 2016).

Current evidence strongly supports a paradigm shift in the diagnosis of PD with a new focus on defining prodromal stages of the disease (Poewe et al. 2017). Because therapeutic interventions should ideally target the triggering pathogenic events as early as possible to achieve not only slowing of disease progression but also forestalling of disease onset, early diagnosis is a key priority and creates an urgent need for valid PD biomarkers with predictive validity for PD diagnosis (Mahlknecht et al. 2015; Poewe et al. 2017). Indeed, there is preliminary evidence that novel MR markers seems to identify prodromal degenerative parkinsonism as loss of DNH was found in at least two-thirds of subjects with idiopathic REM sleep behaviour disorder (iRBD) (De Marzi et al. 2016) and in clinically asymptomatic LRKK2 carriers (Ceravolo et al. 2015). Moreover, a study including patients with PD as well as symptomatic and asymptomatic LRRK2 and Parkin mutation carriers found that R2* values in the SN were increased in PD and mutation-carrying patients as compared with controls and in mutation-carrying patients as compared with PD, while asymptomatic mutation carriers showed higher R2* values than controls and did not differ from PD patients, suggesting that iron deposition occurs early during the preclinical phase of the disease and that R2* measurements may be used as markers for investigating nigrostriatal damage in preclinical mutation-carrying patients (Pyatigorskaya et al. 2015). Although abnormalities described in studies using MTI and MRS/MRSI at 1.5 T lack replication not only at 1.5 T but also at higher field strengths, the increased SNR with its advantages (see “Techniques”) provided by high-field scanning may open a window into providing more robust results in detecting abnormalities in the SN, nigrostriatal pathway and outside the nigrostriatal system using MTI and MRS/MRSI. Not only structural MR marker seems to be altered in prodromal PD as it has been demonstrated by a recent rs-fMRI study in 26 patients with iRBD, 48 patients with PD and 23 healthy control subjects, where connectivity measures of BGN dysfunction differentiated both iRBD and PD from controls with high sensitivity (96%) and specificity (74% for iRBD and 78% for PD), indicating its potential as an indicator of early basal ganglia dysfunction (Rolinski et al. 2016).

An evolving field in image analysis derives from recent advances in image analysis algorithms, which led to the development of novel approaches for automated differentiation of parkinsonian syndromes on single-patient level. These fully automated methods use SVM classification and other machine-learning method-derived classification algorithms for quantitative MRI analysis including volumetric datasets (Huppertz et al. 2016; Scherfler et al. 2016), neuromelanin imaging (Castellanos et al. 2015), diffusion imaging (Haller et al. 2012) and rs-fMRI (Chen et al. 2015). If preliminary results should be confirmed by further large-scaled studies, automated image analysis may open up another window into detecting objectively degenerative parkinsonian disorders on an individual basis in an operator-independent and automated way.

Taken together, further developments and advanced MR imaging techniques could add diagnostic information and could lead to an earlier diagnosis in patients with PD, additionally detecting prodromal stages of PD or distinguishing PD from APDs.

## Abbreviations

1H-MRS: Proton magnetic resonance spectroscopy
AD: Axial (or longitudinal) diffusivity
ADC: Apparent diffusion coefficient
ADCave: Average of ADCs
APD: Atypical parkinsonian disorder
ASL: Arterial spin labelling
AUC: Area under the curve
BGN: Basal ganglia network
CBD: Corticobasal degeneration
CBS: Cortico basal syndrome
Cho: Choline-containing compounds
cMRI: Conventional MRI
Cr: Phosphocreatine
CSF: Cerebrospinal fluid
DIP: Drug-induced parkinsonism
DNH: Dorsolateral nigral hyperintensity
DRTT: Dentatorubrothalamic tract
DTI: Diffusion tensor imaging
DWI: Diffusion-weighted imaging
EPI: Echo-planar imaging
ET: Essential tremor
FA: Fractional anisotropy
FDG-PET: [18-F]-Fluorodeoxyglucose positron emission tomography
FLAIR: Fluid-attenuated inversion recovery
FW: Free-water
GE: Gradient echo
GM: Grey matter
IBZM-SPECT: [132-I]-iodobenzamide-single-photon emission computed tomography
LC: Locus coeruleus
m_a_/p_a_-ratio: Midbrain to pontine area ratio
MCP: Middle cerebellar peduncle
m_d_/p_d_-ratio: Midbrain to pons diameter ratio
MDS: International Parkinson and Movement Disorder Society
MIT: Magnetization transfer imaging
MPRAGE: Magnetization-prepared rapid acquisition with gradient echo
MRI: Magnetic resonance imaging
MRPI: MR parkinsonism index
MRS: Magnetic resonance spectroscopy
MRV: MR volumetry
MSA: Multiple system atrophy
MSA-C: Cerebellar variant of MSA
MSA-P: Parkinsonian variant of MSA
MT: Magnetization transfer
MTR: Magnetization transfer ratio
NAA: *N*-Acetylaspartate
NBIA: Neurodegeneration with brain iron accumulation
NM-MRI: Neuromelanin-sensitive MRI
NODDI: Neurite orientation dispersion and density imaging
PD: Parkinson’s disease
PSP: Progressive supranuclear palsy
QSM: Quantitative susceptibility mapping
RAFF: Relaxations along a fictitious field
RD: Radial (or transverse) diffusivity
ROI: Region of interest
rs-fMRI: Resting-state functional MRI
SCA: Spinocerebellar ataxia
SCP: Superior cerebellar peduncle
SN: Substantia nigra
SNc: SN pars compacta (SNc)
SNr: Substantia nigra pars reticulate
SNR: Signal-to-noise ratio
STEAM: Stimulated echo acquisition mode
STN: Subthalamic nucleus
SVM: Support vector machine
SWI: Susceptibility-weighted imaging
T: Tesla
TBSS: Tract-based spatial statistic
TE: Echo-time
UKPDSBB: United Kingdom Parkinson’s Disease Society Brain Bank
VBM: Voxel-based morphometry
WMC: White matter changes
